# Neurophysiological mechanisms of focused attention meditation: A scoping systematic review

**DOI:** 10.1162/IMAG.a.14

**Published:** 2025-05-28

**Authors:** Jonathan M. Lieberman, Patrick A. McConnell, Mar Estarellas, Matthew D. Sacchet

**Affiliations:** Department of Psychiatry and Behavioural Neurosciences, McMaster University, Ontario, Canada; Department of Psychiatry and Behavioral Neurosciences, University of California, San Francisco, CA, United States; Consciousness and Cognition Lab, University of Cambridge, Cambridge, United Kingdom; School of Biological and Behavioural Sciences, Queen Mary University of London, London, United Kingdom; Meditation Research Program, Department of Psychiatry, Massachusetts General Hospital, Harvard Medical School, Boston, MA, United States

**Keywords:** focused attention meditation, EEG, MEG, scoping systematic review, spectral power

## Abstract

Focused attention meditation (FA) is a foundational and widely studied practice that cultivates sustained concentration by focusing on a specific object, such as the breath, while disengaging from distractions. Numerous studies have investigated the neurophysiological mechanisms of FA, examining aspects such as spectral power, connectivity patterns, and neural entropy. However, despite this extensive research, clarity regarding the methodological approaches and key findings in this field remains limited. This scoping systematic review aimed to collate and interpret key information from electroencephalography (EEG) and magnetoencephalography (MEG) studies on FA, with a focus on study population composition, experimental design, and neurophysiological outcomes. Our findings revealed substantial heterogeneity in participant characteristics, potentially contributing to variability in neurophysiological results, while the choice of FA tasks and control conditions was relatively consistent. In terms of neurophysiological outcomes, consistent trends indicate that FA is associated with increased power in the alpha, beta, and gamma bandwidths, as well as heightened complexity and reduced criticality measures. Based on the findings of this review, we propose several methodological recommendations to improve the quality of future research. Additionally, we identified significant evidence gaps when considering the whole body of research, including the limited use of MEG and a lack of longitudinal studies, pointing to areas for future investigation. Overall, this review provides a firm grounding for the study of the neurophysiology of FA, as well as the study of advanced meditation and neuroscience-informed meditative development.

## 1 Introduction

Meditation—an ancient practice that stems from diverse cultural and religious traditions—comprises a broad spectrum of mind–body techniques designed to enhance attention, awareness, and emotional regulation (Lutz et al., 2008;Sparby & Sacchet, 2022;Wallace, 2005;Walsh & Shapiro, 2006). Over the past few decades, the global popularity of meditation has surged, largely driven by its accessibility and a growing body of scientific evidence highlighting its benefits for mental and physical wellness. Research has demonstrated that meditation can significantly reduce stress and anxiety, alleviate pain, lower blood pressure, decrease the risk of heart disease and stroke, elevate mood and overall well-being, improve focus and cognitive function, enhance sleep quality, and bolster immune function (Arch & Landy, 2015;Black & Slavich, 2016;Brown et al., 2007;Chiesa et al., 2011;Creswell, 2017;Dunning et al., 2019;Galante et al., 2021;Goyal et al., 2014;Hofmann & Gómez, 2017;Pascoe et al., 2017;Rusch et al., 2019;Walsh & Shapiro, 2006).

Meditation can be understood as a family of practices whose effects include regulating attention and emotion, where each specific type of practice involves different psychological processes. These practices are commonly classified in the literature as focused attention, open monitoring, and loving-kindness/compassion meditation (Brandmeyer et al., 2019;Brewer et al., 2011;Cahn & Polich, 2006;Dahl et al., 2015;Fox et al., 2016;D. J. Lee et al., 2018;Lippelt et al., 2014;Lutz et al., 2008,^2015^;Schoenberg & Vago, 2019;Travis & Shear, 2010;Valentine & Sweet, 1999). However, this classification scheme is not exhaustive, and the boundaries between these practices can often be indistinct calling for greater attention to systematizing meditation practices based on phenomenological activities (Sparby & Sacchet, 2022).

Focused attention meditation (FA) is a prevalent practice that involves maintaining a state of selective attention on a specific object, such as the breath, bodily sensations, or visual stimuli, while actively disengaging from distractions (Lutz et al., 2008). This practice results in a narrow aperture of focus characterized by high clarity and stability (Lutz et al., 2008,^2015^). Often considered a dynamic subtype of FA, the body scan is a widely practiced, somatically oriented, attention-focusing technique that commonly serves as an introductory practice in meditation training programs. During a body scan, practitioners typically begin by focusing on their breathing before systematically directing and sustaining attention on different regions of the body in sequence (Dreeben et al., 2013;Gan et al., 2022). Conversely, open monitoring (OM) practices cultivate a state of reflexive awareness with a broad scope of attention, without focusing on a single object (Lutz et al., 2008,^2015^). Constructive meditation practices, such as compassion and loving-kindness meditation, involve generating and sustaining positive feelings towards oneself and others (Dahl et al., 2015). Despite differences between these practices, the regulation of attention remains a critical commonality, specifically involving directing and sustaining attention on the target state, noticing and disengaging from mind wandering and other distractions, and refocusing attention on the target state (Dahl et al., 2015;Lutz et al., 2008,^2015^).

FA is widely recognized as a foundational technique for cultivating the attentional skills necessary for other meditation practices (Dahl et al., 2015;Hölzel et al., 2011;D. J. Lee et al., 2018;Lutz et al., 2008;Travis & Shear, 2010). Due to its straightforward instructions that orient one’s attention to focus on a chosen meditation object, early-stage FA is often accessible to beginning meditators who may not have prior meditation experience. By focusing attention on a single object, such as the breath, and learning to recognize and redirect attention away from distractions, over time FA practice enhances sustained attention and cognitive control—skills that are crucial for other meditation practices. Indeed, many meditation traditions, including Theravada and Tibetan Buddhism (Hart, 1987;Lutz et al., 2007), and Indian Yoga traditions (Devananda, 1999), emphasize the importance of attentional training early in one’s practice. Mastery of FA is also essential for advanced meditation practices which enable access to deep concentrative absorption states (e.g., Jhanas) and other profoundly altered states of consciousness (Ganesan et al., 2024;Sparby & Sacchet, 2022,^2024^;Travis & Parim, 2017;Travis & Shear, 2010;Wright et al., 2023;Yang, Chowdhury, et al., 2024;Yang, Sparby, et al., 2024). Given its role as a foundational technique and its accessibility to novices, FA has become a popular choice among studies investigating the neuroscience of meditation.

Over the past few decades, research on the neural correlates of FA has proliferated, employing neuroimaging techniques such as functional magnetic resonance imaging (fMRI), electroencephalography (EEG), and magnetoencephalography (MEG). fMRI studies have revealed the involvement of specific brain regions and functional connections in FA, typically associated with cognitive processes including conflict monitoring, interoception, and cognitive control (Hölzel et al., 2011;Lutz et al., 2008;Sezer et al., 2022;Treves et al., 2024). A recent systematic review and meta-analysis identified several brain regions within key functional brain networks—the default mode, salience, and executive control networks—as consistently associated with FA (Ganesan et al., 2022). However, fMRI presents challenges and limitations in the study of FA, including limited temporal resolution for tracking brain activity changes, an artificial meditative environment (e.g., loud repetitive noises and restrictive lying position), and high costs that limit longitudinal studies.

Alternatively, a longer tradition of research has investigated the functional role of neural oscillations in meditation using EEG and MEG. These techniques offer insights into fast neural oscillations that are not conventionally detectable through fMRI and thus can provide a more detailed understanding of the temporal dynamics of brain activity related to FA. EEG has been extensively used to characterize specific neurophysiological patterns associated with FA (e.g.,Cahn & Polich, 2006;Fucci et al., 2018,^2022^;Irrmischer et al., 2018;Lomas et al., 2015;Lu & Rodriguez-Larios, 2022;Saggar et al., 2012). MEG, which measures minute magnetic fields that are generated by neural activity (Brookes et al., 2011), offers both high temporal resolution akin to EEG and improvements to spatial resolution. Despite its high cost and technical demands, MEG can be conducted in more naturalistic settings compared with fMRI, reducing artificial constraints that may affect meditation practices. Taken together, EEG and MEG are important tools for elucidating neurophysiological underpinnings of FA, to complement what might be inferred from fMRI alone.

Numerous studies have documented changes in neurophysiological patterns associated with FA, such as increased theta and alpha power (e.g.,Calvetti et al., 2021;Fucci et al., 2018,^2022^;Irrmischer et al., 2018;Lu & Rodriguez-Larios, 2022;Marzetti et al., 2014;Saggar et al., 2012). However, significant variability exists among these studies, including the examination of diverse study populations (e.g., age, sex, participants’ amount and type of meditation training), experimental designs (e.g., type and duration of FA tasks and control conditions), and neurophysiological outcomes (e.g., spectral power, non-linear measures, event-related potentials). This variability complicates the interpretation of these studies and highlights the need for a comprehensive literature review to clarify the scope of existing neurophysiological research on FA. However, to our knowledge, no such review has been conducted.

Rather, previous systematic reviews have either examined the neurophysiological literature related to mindfulness meditation (Chiesa & Serretti, 2010;Lomas et al., 2015) or failed to differentiate between various meditation techniques (Cahn & Polich, 2006), thus impeding the identification of neurophysiological markers uniquely associated with FA. Additionally, none of these previous systematic reviews followed structured processes such as the Preferred Reporting Items for Systematic Reviews and Meta-Analyses (PRISMA) guidelines (Page et al., 2021). Although one review did examine neurophysiological changes across four commonly studied meditation techniques—FA, OM, loving-kindness, and transcendental meditation—it employed a narrative rather than a systematic methodology (D. J. Lee et al., 2018). Moreover, these reviews are now outdated, as many relevant studies have been published in recent years. Taken together, there is a pressing need for an updated review specifically focused on characterizing the neurophysiological research on FA, a foundational and widely studied meditation practice.

### 1.1 Study overview

Despite an abundance of studies investigating the neurophysiological mechanisms of FA, the patterns in methodological approaches and key findings of this body of research remain unclear. Hence, this scoping systematic review aims to provide an updated and comprehensive overview of studies that have used EEG and MEG to explore various aspects of brain function during FA, including spectral power, functional connectivity, and neural entropy. Specifically, this review aims to address the following research questions:

(i) What is the composition of study populations in the literature (e.g., sample size, age, sex, amount and type of meditation training), and how do these factors relate to neurophysiological outcomes during FA?
(ii) What experimental designs are represented in the literature (e.g., type and duration of FA tasks and control conditions) and how do these factors relate to neurophysiological outcomes during FA?
(iii) What neurophysiological outcomes are examined in the FA literature (e.g., spectral power, non-linear measures, event-related potentials)?
(iv) What are the current evidence gaps in the FA literature?

## 2 Methods

We followed the PRISMA-extension checklist for scoping reviews (Page et al., 2021). In the following sections, we describe our eligibility criteria, information sources, search strategy, selection process, and data extraction methods.

### 2.1 Study selection

A search was conducted to identify studies investigating neurophysiological outcomes during FA. An initial search of several databases (Scopus, PubMed, Embase, Web of Science, PsycINFO, and MEDLINE) was conducted on March 15th, 2023. Search terms were derived from the two core concepts in this review, FA and neurophysiology, and informed by a previous systematic review of FA using fMRI (Ganesan et al., 2022). Specifically, FA-related search terms included “focused attention,” “focused breathing,” “breath awareness,” “internal attention,” “interoceptive attention,” “concentration,” “Samatha,” “Trataka,” “Zen,” “Anapanasati.” These were searched in conjunction with neurophysiology-related terms which included “EEG,” “electroencephalography,” “MEG,” and “magnetoencephalography.” We also searched through the reference lists of all included studies and other relevant literature reviews (i.e.,Cahn & Polich, 2006;D. J. Lee et al., 2018;Lomas et al., 2015) to ensure that no studies were missed. Before finalizing data analysis, the same search was repeated on December 13th, 2023, and again on August 12th, 2024, to include studies that were published after the initial search date up until the first manuscript submission.

All articles identified by the search were imported into Covidence (https://www.covidence.org/), a web-based collaboration software platform that streamlines the process of completing systematic and other literature reviews. Following PRISMA guidelines, JML and ME independently screened articles against defined inclusion and exclusion criteria, initially based on title and abstract reviews, followed by full-text reviews. Inclusion criteria were (i) peer-reviewed and empirical (including case studies), (ii) measurement of brain activity using EEG or MEG during FA in a fixed posture, and (iii) written in English. Exclusion criteria were (i) non-peer-reviewed or non-empirical (e.g., conference abstract, book chapter, dissertation, preprint); (ii) measurement of brain activity using modalities other than EEG or MEG (e.g., fMRI, fNIRS, PET); (iii) the use of meditation techniques other than fixed FA (e.g., OM, mindfulness, compassion, transcendental, body scans); (iv) measurement of brain activity only before and/or after (but not during) FA; (v) the use of bio/neurofeedback during FA; (vi) the use of breathwork techniques that represent substantial alterations in typical breathing patterns (e.g., deep abdominal breathing); and (vii) the use of movement-based techniques that involve focused attention (e.g., Qi-gong, Tai Chi, yoga). Although some breathwork or movement-based techniques may be classified as types of FA, they were excluded as they are likely to induce additional confounding factors on neurophysiological signals.

Given the significant variability in terminology across meditation traditions and in the literature, the determination of whether a particular meditation technique constituted FA was based on each study’s description of the technique and/or the specific instructions provided to participants. In many cases, multiple meditation techniques were examined within the same study. These studies were only included in the review when results were specifically delineated for FA alone. Additionally, some advanced meditative states, such as the Jhanas, are accessed and navigated by applying FA in the initial stages and throughout (for review,Yang, Sparby, et al., 2024). Studies incorporating these techniques were only included when the results clearly distinguished between FA and the more advanced states. For example, among the three existing EEG studies examining Jhana meditation (DeLosAngeles et al., 2016;Dennison, 2019;Hagerty et al., 2013), only one was included for this reason (Hagerty et al., 2013). In cases of disagreements between reviewers (JML, ME) regarding inclusion versus exclusion, a third author (PAM) made a final decision after a discussion with the initial reviewers. Articles that were excluded during the full-text review were tagged with primary exclusion reasons to facilitate PRISMA flow chart reporting.

### 2.2 Data extraction

Data from the included studies were systematically extracted and arranged in spreadsheets. Extracted data were broadly classified into four categories: (i) study characteristics (e.g., authors, publication year, data availability, experimental design); (ii) participant characteristics (e.g., sample size, sex, age, handedness, meditation experience); (iii) data collection (e.g., target of focus in FA task, environmental conditions for FA task, instructions given to participants, length of FA task, type and length of control condition, the total number of FA task and control condition blocks, number of runs/sessions, data acquisition, and processing methods); and (iv) neurophysiological outcomes (e.g., frequency bands, spectral power, and coherence, non-linear measures, event-related potential components).

In this review, participants were categorized into three groups: meditative-naïve (i.e., individuals with negligible or no prior meditation experience), novice meditators, and long-term meditators. Due to significant variability in how studies reported meditation experience, defining strict cutoff criteria (e.g., based on meditation hours) was not feasible. Instead, classifications were based on a combination of reported meditation experience, study author descriptions, participant labels, and recruitment sources. Among studies that conducted comparisons between groups of meditators with differing amounts of previous experience, the less experienced group was categorized as novice meditators and the more experienced group as long-term meditators. In addition to neurophysiological outcomes associated with FA, some eligible manuscripts also reported outcomes pertaining to other meditation techniques (e.g., OM, loving-kindness), which were not extracted as they fall outside the scope of this review. We only extracted results that were reported as statistically significant according to the criteria implemented in each manuscript.

## 3 Results

### 3.1 Search results

**Fig. 1.**
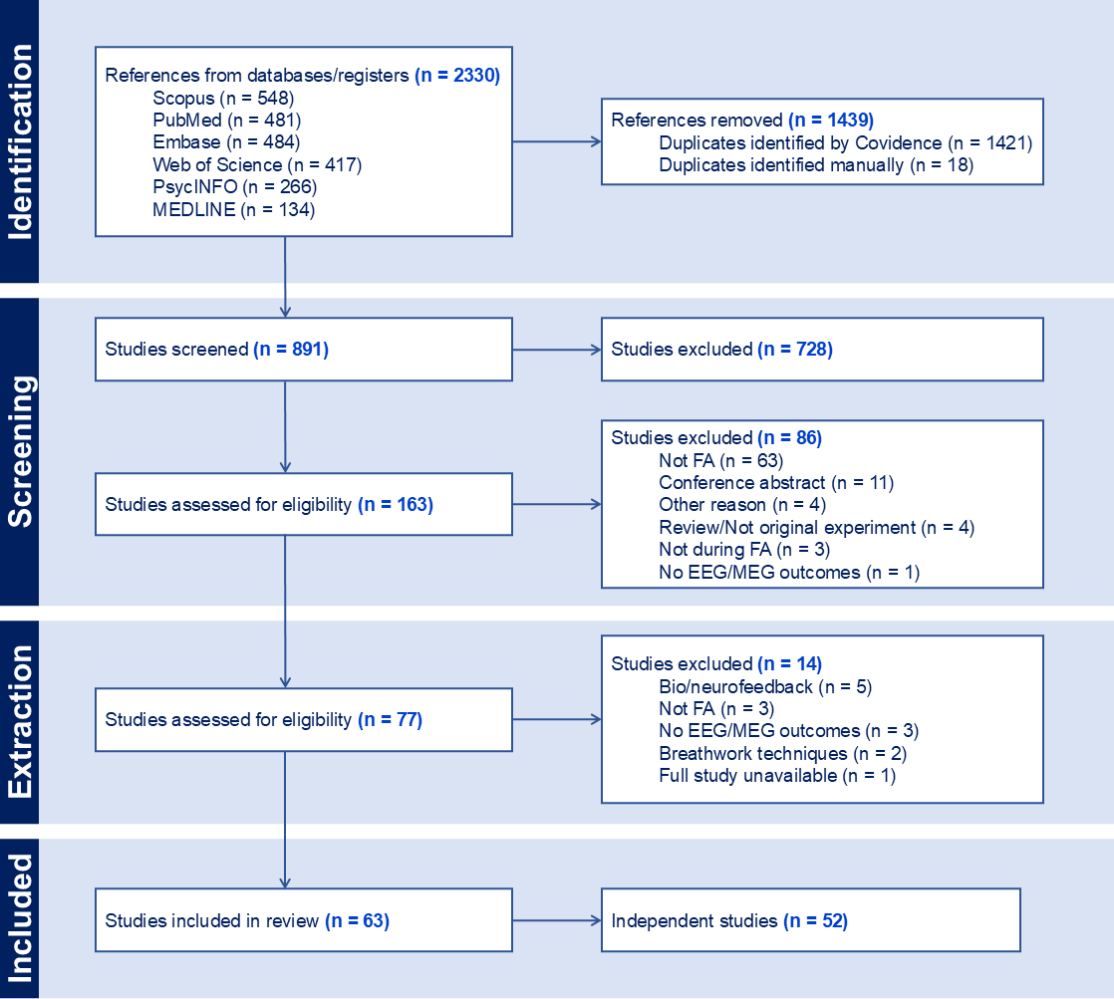
PRISMA flowchart illustrating the results of the scoping review study procedure. FA = focused attention meditation, EEG = electroencephalography, MEG = magnetoencephalography.

A total of 2,330 manuscripts were initially imported for screening. After removing duplicate citations (n = 1,439), 891 potentially relevant manuscripts remained. From the abstract review, 728 manuscripts were excluded. During the full-text review, 86 manuscripts were excluded. Additionally, 14 manuscripts were excluded during data extraction. Thus, a total of 63 manuscripts were eligible for inclusion in this review. Among these, 12 manuscripts conducted secondary analyses using datasets whose original manuscripts were also included in the review (Calvetti et al., 2021;Dadhich et al., 2024;D’Andrea et al., 2024;Lin, White, Viravan, et al., 2024;Lu & Rodriguez-Larios, 2022;Martínez Vivot et al., 2020;Pandey et al., 2022;Sihn et al., 2024;Takahashi et al., 2005;Ventura et al., 2024;Walter & Hinterberger, 2022;Yordanova et al., 2021), while 1 manuscript included two separate studies with independent samples (Irrmischer et al., 2018). Therefore, the 63 manuscripts included in this review represent results from 52 independent studies (Fig. 1). In the subsequent two results sections (*3.1 Participant characteristics*;*3.2 Experimental design: FA tasks and control conditions*), methodological data from manuscripts conducting secondary analyses were excluded to avoid double counting. In the results section following those (*3.3 Neurophysiological results*), neurophysiological outcome data from all 63 eligible manuscripts (including secondary analyses) were included.

### 3.2 Participant characteristics

Table 1provides an overview of study population composition across the 52 independent studies included in this review. In total, there were 1,453 participants (mean = 27.9; SD = ± 16.8), with the number of participants per study ranging from 1 to 68. The average age of participants across studies was 37.5 years (SD = ± 12.4), with individual study averages ranging from 19.3 to 67.2. There were fewer females (627; 43.2%) than males (826; 56.8%) across all studies. Most studies did not report the ethnicities (44/52; 84.6%) or handedness (35/52; 67.3%) of participants. Nearly all studies (49/52; 94.2%) included only healthy participants, defined as those without any reported clinical symptoms or conditions.

**Table 1.** Study population composition.

Author(s)	Year	Participant group(s)	Sample size, N (female)	Age in years, mean ± S.D or range	Amount of meditation training in H or Y, mean ± S.D, minimum, or range	Type of FA-M training
Fucci	2022	naïve & long-term	naïve: 36 (17) long-term: 30 (13)	naïve: 52.0 ± 7.6 long-term: 52.0 ± 7.8	>10,000 H	Tibetan Buddhism
Hunkin	2021	Mixed	68 (40)	22.7 ± 7.4	0.25–19 Y ^1^	NR
Yordanova	2020	long-term only	22 (4)	44.2	19,358 ± 3,164 H	Theravada Buddhism
van Son	2019	not stated	26 (26)	22.8 ± 2.6	NR	NR
Fucci	2018	naïve & long-term	naïve: 15 (2) long-term: 16 (4)	naïve: 42.4 ± 11.4 long-term: 43.4 ± 9.4	28,990 ± 13,880 H	Tibetan Buddhism
Hinterberger	2014	long-term only	30 (11)	47.0	6,498 H	Varied
Saggar	2012	long-term only	44 (23)	46.9	2,564 H	Samatha
Marzetti	2014	long-term only	8 (0)	37.9 ± 9.4	15,750 H	Theravada Buddhism
Coomans	2021	not stated	22 (12)	22.3 ± 2.9	NR	NR
Rodriguez-Larios	2021	naïve & long-term	naïve: 29 (15) long-term: 29 (17)	naïve: 47.1 ± 13.9 long-term: 47.3 ± 11.2	9.8 ± 7.1 Y	Varied
Kakumanu	2018	novice & long-term ^2^	novice: 24 (12) long-term ( *sen* ): 22 (11) long-term ( *tea* ): 21 (10)	novice: 48.4 ± 10.1 long-term ( *sen* ): 54.2 (12.6) long-term ( *tea* ): 51.8 (12.2)	novice: 1,080 ± 600 H long-term ( *sen* ): 10,364 ± 5,229 H long-term ( *tea* ): 15,349 ± 9,307 H	Vipassana
Brandmeyer & Delorme	2018	novice & long-term	novice: 12 (10) long-term: 12 (3)	novice: 45.0 ± 14.8 long-term: 39.3 ± 12.0	novice: 3.2 ± 3.1 H/week long-term: 14.8 ± 1.6 H/week	Himalayan Yoga
Milz	2014	naïve only	23 (0)	23.2 ± 1.9	N/A	N/A
Tsai	2013	novice only	1 (0)	44.0	2 Y	Breath focus
Lavallee	2011	naïve & novice	naïve: 7 (3) novice: 7 (4)	naïve: 24.7 ± 1.9 novice: 25.4 ± 1.8	215.9 ± 29.0 H	Concentration
Baijal & Srinivasan	2010	naïve & long-term	naïve: 10 (4) long-term: 10 (3)	naïve: 35.0 ± 5.0 long-term: 39.0 ± 5.0	3–7 Y	Sahaj Samadhi
Murata	2004	naïve only	22 (0)	22.3 ± 2.1	N/A	N/A
Kubota	2001	naïve only	12 (6)	24.3	N/A	N/A
Corby	1978	naïve & novice & long-term	naïve: 10 (4) novice: 10 (4) long-term: 10 (4)	naïve: 22.9 novice: 23.7 long-term: 25.8	novice: 2,376 H long-term: 5,460 H	Ananda Marga
Dunn	1999	novice only	9 (NR)	NR	45.1 H	Concentration and mindfulness
Chotipanich	2021	mixed	6 (4)	50.5	0.083–50 Y	Anapanasati
Sharma	2022	mixed	34 (22)	18–68	0–1+ Y	NR
Park & Park	2012	naïve only	58 (22)	24.7	N/A	N/A
Duda	2023	naïve only	33 (17)	23.5 ± 4.5	N/A	N/A
Pal	2022	long-term only	15 (0)	25.0 ± 4.9	5.0 ± 3.6 Y	Yoga (Dharana)
Hagerty	2013	long-term only	1 (0)	53.0	6,000 H	Jhana
Park	2021	naïve only	30 (15)	28.1	N/A	N/A
Braboszcz	2017	naïve & long-term	naïve: 16 (5) long-term: 48 (9)	naïve: 45.0 ± 10.0 long-term: 45.3	9,100 H	Vipassana, Himalayan Yoga, Isha Yoga
Steinhubl	2015	naïve & novice	naïve: 20 (16) novice: 20 (16)	naïve: 53.5 (11.2) novice: 48.9 (11.9)	0.53 ± 0.15 H/day	NR
Colgan	2019	novice only	1 (0)	32.0	2,200 H	Vipassana, Mindfulness-based Stress Reduction
Amihai & Kozhevnikov	2014	long-term only	19 (3)	44.3	7.7 Y	Theravada, Vajrayana
Jo	2019	naïve & long-term	naïve: 11 (9) long-term: 9 (5)	naïve: 40.2 ± 11.4 long-term: 49.7 ± 7.5	22.0 ± 7.96 Y	Varied
Elson	1977	naïve & novice	naïve: 10 (3) novice: 10 (3)	NR	1.6 Y ^3^	Ananda Marga
Young	2021	long-term only	28 (14)	52.3 ± 15.7	21,935 ± 20,186 H	Varied
Matiz	2021	novice only	32 (19)	43.7 ± 12.2	2.3 ± 1.6 Y	Mindfulness
Kopal	2014	naïve & novice	naïve: 7 (1) novice: 7 (0)	naïve: 20–50 novice: 20–40	>1,000 H	Theravada Buddhism
López	2022	mixed	12 (8)	34.9 ± 11.9	0–8+ Y	NR
Telles	2015	novice only	48 (0)	19.3 ± 2.6	>318 H	Mantra
Rodriguez-Larios & Alaerts	2021	naïve only	28 (17)	23.5	N/A	N/A
Lee	2017	naïve & novice & long-term	naïve: 10 (7) novice: 10 (5) long-term: 10 (3)	naïve: 54.0 ± 9.4 novice: 49.1 ± 6.4 long-term: 54.5 ± 15.4	novice: 6.0 ± 4.5 Y long-term: 17.1 ± 8.8 Y	Tibetan Nyingmapa
Atchley	2016	naïve & novice & long-term	naïve: 13 (9) novice: 15 (11) long-term: 14 (6)	naïve: 48.0 ± 11.0 novice: 50.0 ± 13.0 long-term: 49.0 ± 15.0	novice: <1,000 H, 2.4 ± 2.5 Y long-term: >4,000 H, 22.6 ± 13.2 Y	Novice: Mixed secular practices Long-term:Tibetan or Zen Buddhism
Śliwowski	2023	naïve only	16 (10)	23.3 ± 2.2	N/A	N/A
Lazarou	2023	not stated	40 (30)	67.2	NR	NR
Biedermann	2016	naïve & long-term	naïve: 14 (12) long-term: 12 (5)	naïve: 52.6 ± 15.8 long-term: 55.8 ± 13.6	10–35 Y	Zen, Chan, Tibetan Mahayana
Thomas	2014	novice & long-term	novice: 6 (3) long-term: 6 (3)	novice: 42.0 ± 8.0 long-term: 54.0 ± 6.5	novice: 1,500 H long-term: 11,000 H	Satyananda Yoga
Braboszcz & Delorme	2011	not stated	16 (8)	27.0 ± 5.0	NR	NR
Irrmischer ^4^	2018	naïve & novice	naïve: 11 (9) novice: 8 (4)	naïve: 21.6 ± 2.1 novice: 41.7 ± 6.7	18.0 ± 10.7 Y	Zen, Vipassana
Irrmischer ^5^	2018	naïve & novice	naïve: 10 (5) novice: 20 (10)	naïve: 41.4 ± 14.4 novice: 47.0 ± 12.5	1,247 H	Varied
Rodriguez-Larios	2024	naïve & novice	naïve: 20 (NR) novice: 21 (NR)	NR	0.17 Y	Mindfulness-based Stress Reduction
Heinilä	2024	mixed	29 (NR)	21–48	0–10 Y ^6^	NR
Lin	2024	naïve only	29 (17)	20.7 ± 4.0	N/A	N/A
Neri	2024	mixed	23 (0)	45.7	<1–10+ Y	Tibetan Buddhism

Note: This table provides an overview of the study population composition for all 52 independent studies included in the review.^1^Only 32% of participants reported routine meditation experience, with practice durations ranging from 3 months to 3 years, and one participant having 19 years of meditation experience. The remaining participants were meditation-naïve.^2^In this study, there were two separate groups of long-term meditators—senior practitioners (sen) and meditation teachers (tea).^3^Nine out of 10 novice participants contributed to the average meditation experience, with 1 additional participant being a yogic monk whose years of experience was not reported.^4^Corresponds to the first independent study reported inIrrmischer et al. (2018).^5^Corresponds to the second independent study reported inIrrmischer et al. (2018).^6^13 of 29 participants were meditation-naïve, while 16 of 29 participants had previous meditation experience ranging from 0.5 to 10 years (mean = 3.4 ± 2.4 years). N = number, H = hours, Y = years, S.D = standard deviation, NR = not reported, N/A = not applicable, sen = senior practitioners, tea = meditation teachers.

**Fig. 2.**
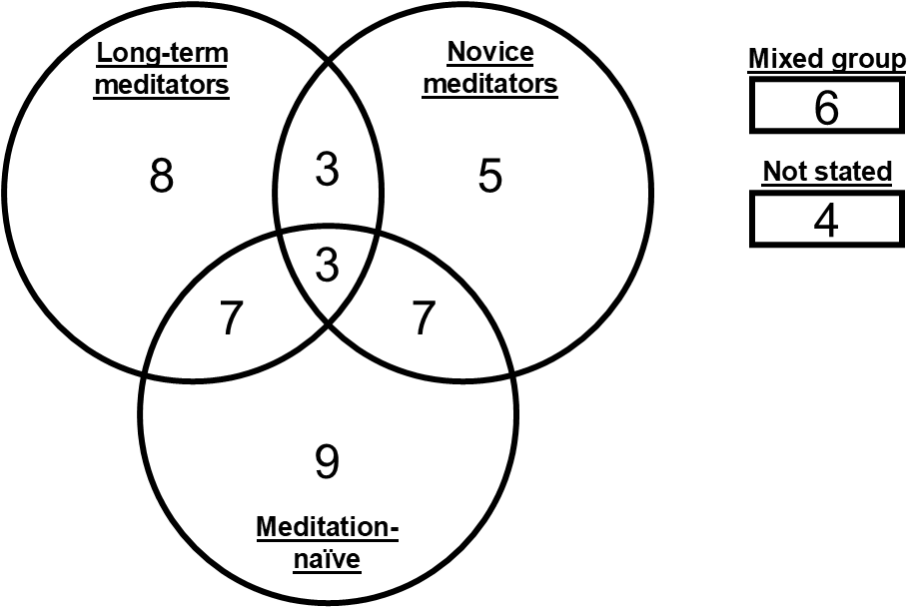
Venn diagram illustrating the distribution of participant groups across the 52 independent studies. The diagram shows the number of studies that included meditation-naïve participants, novice meditators, long-term meditators, and combinations thereof. Each circle represents a different participant group, with overlaps indicating studies that included multiple groups. Separate boxes indicate the number of studies with mixed participant groups and those that did not state the participants’ level of meditation experience.

Meditation-naïve participants (i.e., individuals with negligible or no previous meditation experience) comprised 34.4% of the total sample (N = 500; mean = 19.2; SD = ± 11.4; 26/52 studies). Novice meditators comprised 18.0% of the total sample (N = 261; mean = 14.5; SD = ± 11.3; 18/52 studies) with a cumulative average meditation experience of 1,122.2 hours (SD = ± 888.8), based on a subset of studies (8/18; 44.4%) that either reported these data directly or provided sufficient information to permit an indirect estimate. Long-term meditators comprised 28.6% of the total sample (N = 416; mean = 18.9; SD = ± 11.7; 20/52 studies) with a cumulative average meditation experience of 12,697.4 hours (SD = ± 7,810.8), based on a subset of studies (12/20; 60.0%) that either directly or indirectly reported these data. The remaining 19.0% of the sample included participants with unspecified (N = 104; mean = 26.0; SD = ± 8.8; 4/52 studies) or mixed levels of meditation experience (N = 172; mean = 28.7; SD = ± 20.0; 6/52 studies). The proportion of female participants varied substantially across participant groups, with females constituting a majority of the unspecified (73.1%) group compared with a minority in the mixed (43.0%), naïve (45.0%), novice (38.7%), and long-term (36.3%) groups. Some studies included only a single participant group, while others included multiple participant groups in various combinations (Fig. 2).

### 3.3 Experimental design: FA tasks and control conditions

**Fig. 3.**
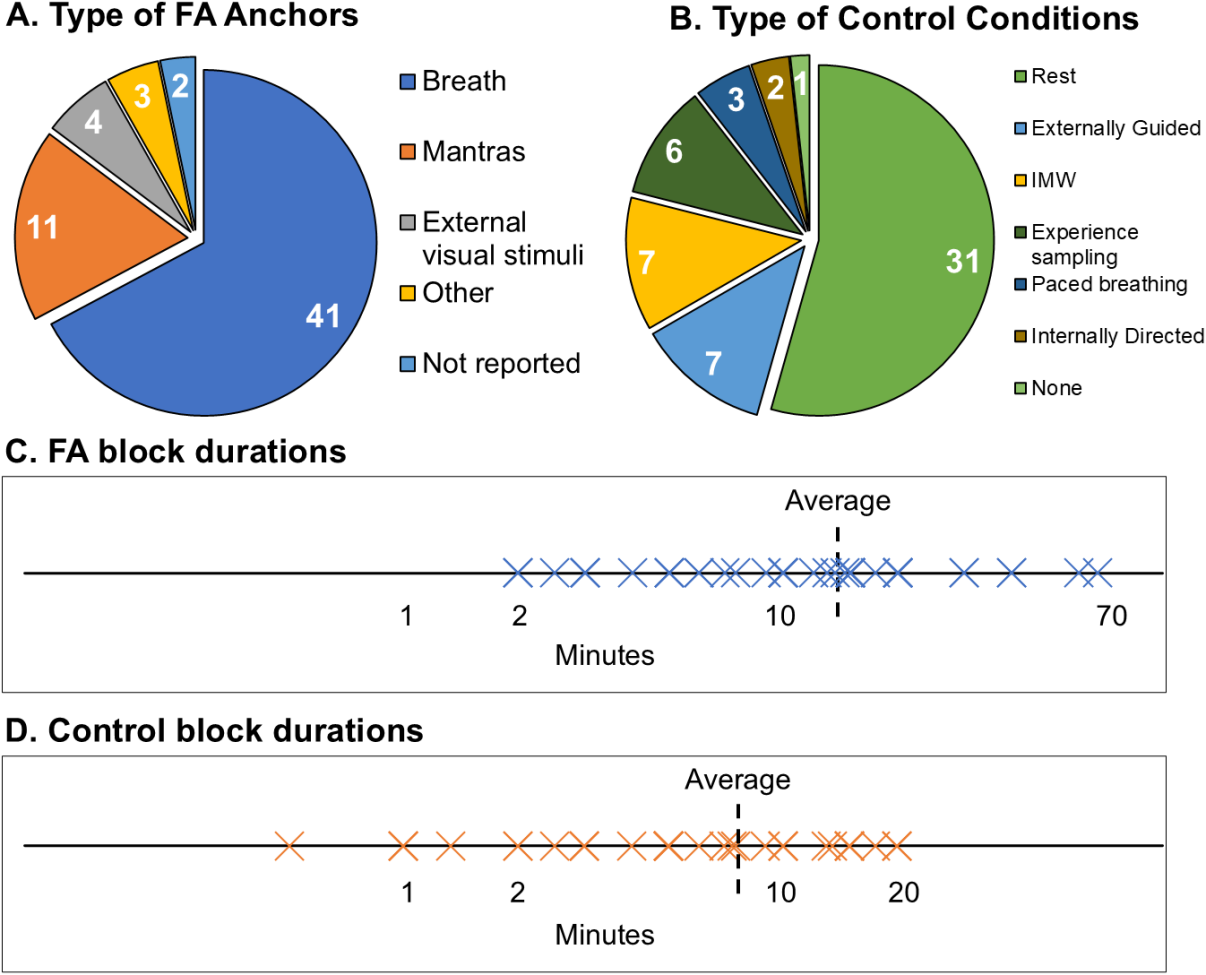
Overview of FA task characteristics and control conditions across the 52 independent studies. (A) Pie chart showing the distribution of different types of anchors used during FA tasks. (B) Pie chart depicting the various control conditions employed in the studies. (C) Log scale line plot with blue crosses indicating the duration of FA blocks for each study (average = 14.8; SD = ± 13.2). (D) Log scale line plot with orange crosses indicating the duration of control condition blocks for each study (average = 7.7; SD = ± 5.8). An average duration was calculated for studies using multiple blocks of different durations. For studies reporting only duration ranges, the middle of the range was used. FA = focused attention meditation, IMW = instructed mind wandering.

Studies varied in the number and type of meditative anchors used during FA tasks. Of the 52 independent studies, 42 used a single anchor, 8 used multiple anchors, and 2 did not specify the anchor. The most common anchor was breath sensations (41 studies), with 11 of those using breath counting. Other anchors included mantras (11), external visual stimuli (4), internal visual imagery (1), body-part sensations (1), and Jhanas (1) (Fig. 3A). Most studies instructed participants to meditate with their eyes closed (43 studies), while fewer used eyes open (6), a combination of open and closed (1), or did not report the eye state (2). FA block durations ranged from 2 to 67.5 minutes, averaging 14.8 minutes (SD = ± 13.2) (Fig. 3C).

The most common control condition was rest (31 studies), followed by externally guided perceptual tasks (7)—such as reading (Fucci et al., 2018;Hinterberger et al., 2014;Hunkin et al., 2021), listening to a podcast (Śliwowski et al., 2023), or TED talk (Lin, White, Viravan, et al., 2024), viewing a movie (Fucci et al., 2022), or tone identification (Atchley et al., 2016). Other control conditions included instructed mind wandering (7), paced breathing (3), and internally directed cognitive tasks (2)—an imagination exercise (i.e., “think about how to build a tree house”) (Biedermann et al., 2016) and mental arithmetic (Thomas et al., 2014) (Fig. 3B). While most studies used a single control condition, there were three studies that used multiple control conditions (Hinterberger et al., 2014;Śliwowski et al., 2023;Telles et al., 2015) and one study that did not include any control condition (Sharma et al., 2022). Notably, there were also six studies that used experience sampling paradigms to compare focused attention versus naturally occurring mind wandering (Braboszcz & Delorme, 2011;Brandmeyer & Delorme, 2018;Rodriguez-Larios et al., 2021,^2024^;Rodriguez-Larios & Alaerts, 2021;van Son et al., 2019). Control durations ranged from 0.5 to 20 minutes, averaging 7.7 minutes (SD = ± 5.8) (Fig. 3D).

### 3.4 Neurophysiological outcomes

Across the 63 eligible manuscripts, a wide range of neurophysiological outcome types were reported, including spectral analyses (45 studies), non-linear measures (7), event-related potentials (ERPs) (8), machine-learning classification methods (6), consumer-grade EEG (4), and hyperscanning (2) (Table 2). These categories are not mutually exclusive, as several manuscripts reported multiple outcome types. In the following subsections, we provide an overview of all relevant neurophysiological results by outcome type.

**Table 2.** Outcome types of included studies.

Outcome type	Number of studies
Spectral analyses	45
Non-linear measures	7
ERPs	8
Classification methods	6
Consumer-grade EEG	4
Hyperscanning	2

Note: This table indicates the number of studies reporting findings related to each outcome type. These categories are not mutually exclusive, as several studies reported multiple outcome types. ERPs = event-related potentials; EEG = electroencephalography.

#### 3.4.1 Spectral analyses

**Fig. 4.**
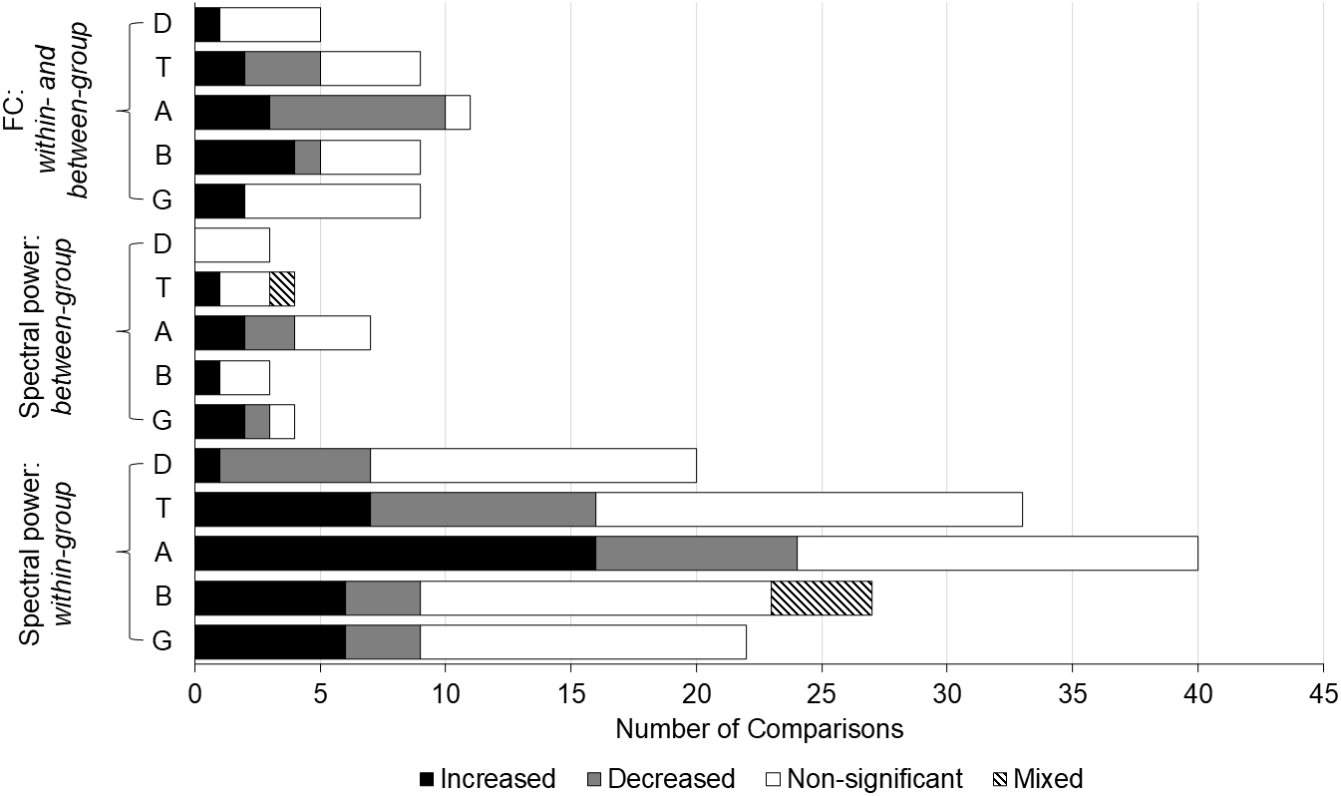
Spectral analysis results by outcome/comparison type and frequency band. This bar graph shows the spectral analysis results categorized by outcome/comparison type (spectral power: within-group, spectral power: between-group, and functional connectivity) and frequency band (Delta, Theta, Alpha, Beta, Gamma). The x-axis represents the number of comparisons across studies for each outcome/comparison type and frequency band. The shaded segments of each bar indicate the proportion of comparisons that yielded increased, decreased, non-significant, or mixed results. The direction of significant results is displayed in relation to comparisons of FA versus other conditions (e.g., rest, control task, other meditation techniques) for within-group comparisons, and more experienced versus less experienced meditators (e.g., long-term meditators vs. novice meditators, novice meditators vs. meditation-naïve participants) for between-group comparisons. Functional connectivity results are combined across within- and between-group comparisons as there was only one between-group comparison of functional connectivity across all included studies. FC = functional connectivity, D = delta, T = theta, A = alpha, B = beta, G = gamma.

Spectral analyses were the most frequently reported neurophysiological outcome type among the eligible manuscripts (45 studies). These studies primarily examined spectral power and functional connectivity across Delta, Theta, Alpha, Beta, and Gamma frequency bands. Findings included both within-group (e.g., FA vs. rest, control conditions, or other meditation techniques) and between-group comparisons (e.g., long-term vs. novice meditators) (Fig. 4). Detailed results for each frequency band are provided in the subsections below. Additional unique outcomes related to these frequency bands, such as correlations with behavioural measures, principal component analysis (PCA), individual alpha frequency (IAF), and band ratio measures, are summarized in a separate subsection. A comprehensive summary of all spectral analysis outcomes by study is provided inTable 3.

**Table 3.** Spectral analysis results.

		Experimental design			Frequency band						
Study	Comparison type	Participant group(s)	Condition(s)	Outcome measure(s)	G	B	A	T	D	Brain region(s)	Other findings
Fucci et al. (2022)	Within (control)	long-term (TB), naïve	FA-M (fc) vs. movie	Absolute power for Alpha *frontal and occipital ROIs	-	-	↑	-	-	Alpha: frontal & occipital	
	Within (meditation)		FA-M (fc) vs. OM	Absolute power for Alpha *frontal and occipital ROIs	-	-	↓	-	-	Alpha: frontal & occipital	
	Between	long-term (TB) vs. naïve	FA-M (fc) vs. OM	Absolute power for Alpha *frontal and occipital ROIs	-	-	ns	-	-	N/A	
Fucci et al. (2018)	Within (control)	long-term (TB), naïve	FA-M (fc) vs. reading	Absolute power for Gamma, Alpha *frontal and occipital ROIs	↓	-	↑	-	-	Alpha: frontal; Gamma: occipital	
	Within (meditation)		FA-M (fc) vs. OM	Absolute power for Gamma, Alpha *frontal and occipital ROIs	ns	-	↓	-	-	Alpha: frontal	
	Between	long-term (TB) vs. naïve	FA-M (fc)	Absolute power for Gamma, Alpha *frontal and occipital ROIs	↑	-	ns	-	-	Gamma: frontal	
López et al. (2022)	Other	mixed (NR)	FA-M (b)	Correlation between Meditative State Scale (MSS) score and Gamma power *12 ROIs	-	-	-	-	-	Gamma: left central parietal, left parieto-occpital, medial frontal cortex, medial frontal central, medial central parietal	Positive correlation between the subfactor “mental quieting” of the MSS and increased gamma power.
Braboszcz et al. (2017)	Within (control)	long-term (HY)	FA-M (m) vs. IMW	Absolute power for Gamma, Alpha *64 electrodes	ns	-	ns	-	-	N/A	
	Within (meditation)	long-term (IS)	FA-M (b) vs. OM	Absolute power for Gamma, Alpha *64 electrodes	↑	-	ns	-	-	Gamma: fronto-central	
	Between	long-term (HY) vs. naïve	FA-M (b/m), FA-M (b)	Absolute power for Gamma, Alpha; Gamma:Alpha ratio *64 electrodes	↑	-	ns	-	-	Gamma: frontal, midline, occipital	Increased gamma:alpha power ratio
	Between	long-term (HY) vs. long-term (V)	FA-M (b/m), FA-M/OM	Absolute power for Gamma, Alpha *64 electrodes	ns	-	↓	-	-	Alpha: global	
Park & Park (2012)	Within (control)	naïve	FA-M (b) vs. ECR	Absolute power for Beta, Alpha, Theta *F3, F4, P3, P4, T3, T4 electrodes	-	↑	↑	↓	-	Beta: right parietal; Alpha: global; Theta: left frontal, left parietal, right temporal	
Park (2021)	Within (control)	naïve	FA-M (bc) vs. PB	Absolute power for Beta, Alpha, Theta *F3, F4, P3, P4, T3, T4 electrodes	-	ns	ns	ns	-	N/A	
Brandmeyer & Delorme (2018)	Within (MW)	long-term (HY)	FA-M (m) vs. MW - *experience sampling*	Absolute power for Alpha, Theta *64 electrodes	-	-	↑	↑	-	Alpha: somatosensory cortex; Theta: frontal	For each subject, there was a positive correlation between the theta and alpha differences between conditions.
	Within (MW)	novice (HY)	FA-M (m) vs. MW - *experience sampling*	Absolute power for Alpha, Theta *64 electrodes	-	-	ns	ns	-	N/A	
Tsai et al. (2013)	Within (rest)	novice (NR) *	FA-M (b) vs. ECR	Absolute power for Alpha, Theta *FP1, FP2	-	-	ns	↑	-	Theta: bilateral frontal	
	Within (meditation)		FA-M (b) vs. EM	Absolute power for Alpha, Theta *FP1, FP2	-	-	↓	↓	-	Alpha, Theta: bilateral frontal	
Corby et al. (1978)	Between	long-term (AM) vs. naïve	FA-M (b/m)	Absolute power for Alpha, Theta *not reported	-	-	↑	↑	-	Not reported	
Chotipanich et al. (2021)	Within (rest)	mixed (Ana)	FA-M (b) vs. rest	Absolute power for Alpha *32 electrodes	-	-	↑	-	-	Alpha: frontal	
Rodriguez-Larios & Alaerts (2021)	Within (MW)	naïve	FA-M (b) vs. MW - *experience sampling*	Absolute and relative power for Alpha, Theta *all 22 electrodes	-	-	↑	↓ ^6^	-	Alpha, Theta: global	Increased mean theta frequency; decreased mean alpha frequency; decreased cross-frequency dynamics (i.e., harmonicity and phase synchrony) between alpha and theta rhythms.
Kubota et al. (2001)	Within (control)	naïve	FA-M (bc) vs. PB	Absolute power for Theta *Fz electrode	-	-	-	↑	-	Theta: frontal midline	12/25 (48%) of participants showed increased frontal midline theta activity.
Jo et al. (2019)	Other	long-term (varied)	FA-M (b) vs. ECR	SCP phase synchrony *19 electrodes	-	-	-	-	-	N/A	Decreased SCP phase synchrony
	Other	naïve	FA-M (b) vs. ECR	SCP phase synchrony *19 electrodes	-	-	-	-	-	N/A	No difference (baseline), Decreased SCP phase synchrony (after completion of 8-week MBSR program)
Elson et al. (1977)	Other	novice (AM), naïve	FA-M (m), ECR	Alpha-Theta state ^1^	-	-	-	-	-	N/A	FA-M: Proportion of time spent in the “alpha-theta” state was consistent around 75%ECR: Proportion of time spent in the “alpha-theta” state dropped from 76% to less than 20% before gradually recovering
Walter & Hinterberger (2022) ^2^	Within (rest)	long-term (varied)	FA-M (fh) vs. ECR	Absolute power for Gamma, Beta, Alpha, Theta, Delta *64 electrodes	↑	ns	ns	↓	↓	Gamma, Theta, Delta: global	
	Within (meditation)		FA-M (fh) vs. EM	Absolute power for Gamma, Beta, Alpha, Theta, Delta *64 electrodes	↑	ns	ns	ns	ns	Gamma: global	
	Within (meditation)		FA-M (fh) vs. PM	Absolute power for Gamma, Beta, Alpha, Theta, Delta *64 electrodes	ns	ns	↓	ns	ns	Alpha: global	
Yordanova et al. (2020)	Coherence/connectivity	long-term (TVB)	FA-M (b) vs. ECR	Imaginary part of coherence for Beta, Alpha, Theta, Delta *10 regional clusters of electrode pairs	-	↑	↑	↑	↑	Beta: right hemisphere; Alpha: right hemisphere with parieto-occipital cluster; Theta: left hemisphere with posterior integrating focus; Delta: global	Increased correlation between left-hemispheric theta and right-hemispheric alpha connectivity.
Martínez Vivot et al. (2020) ^2^	Coherence/connectivity	long-term (HY) vs. naïve	FA-M (b)	Coherence for Gamma *64 electrodes	↑	-	-	-	-	Gamma: global	
van Son et al. (2019)	Within (MW)	not stated	FA-M (bc) vs. MW - *experience sampling*	Absolute power for Beta, Alpha, Theta, Delta; Theta:Beta ratio *31 electrodes	-	↑	↑	↓	↓	Not reported.	Decreased frontal midline theta:beta ratio, particularly in posterior midline regions.
Hinterberger et al. (2014)	Within (meditation)	long-term (varied)	FA-M (fh) vs. EM	Absolute power for Gamma, Beta, Alpha, Theta, Delta *13 ROIs across 64 electrodes; global field power ^3^	↑	ns	ns	ns	ns	Gamma: central and parietal brain regions.	Increased gamma was more pronounced for the most long-term meditators in the study; n.s. differences for global field power.
Saggar et al. (2012)	Within (longitudinal)	long-term (Sha) mid- & post-retreat vs. pre-retreat	FA-M (b)	Absolute power for Gamma, Beta, Alpha, Theta, Delta; IAF *73 electrodes	ns	↓	ns	ns	ns	Beta: bilateral medial prefrontal, central and parietal brain regions.	Decreased IAF; the change in IAF was negatively related to daily FA-M practice (i.e., the more participants practiced FA-M on retreat, the more IAF decreased).
Marzetti et al. (2014)	Coherence/connectivity	long-term (TVB)	FA-M (NR) vs. ECR	Functional connectivity of the PCC for Gamma, Beta, Alpha, Theta, Delta *165 channels (MEG)	ns	ns	↓	ns	ns	Alpha: left and right SFG, left SMFG, left LTC, left and right ACC.	
	Coherence/connectivity		FA-M (b) vs. OM	Functional connectivity of the PCC for Gamma, Beta, Alpha, Theta, Delta *165 channels (MEG)	ns	ns	↓	ns	ns	Alpha: left mPFC, left SFG, left dlPFC, left and right ACC, and left IPL.	
Rodriguez-Larios et al. (2021)	Other	long-term (varied)	FA-M (b) vs. ECR	Absolute power (2–30 Hz) without a priori definition of frequency bands, 1/f slope, IAP, IAF *19 electrodes	-	-	-	-	-	N/A	Decreased global power in alpha/beta range (9–30 Hz); decreased IAF (frontal), decreased IAP (parietal).
	Other	naïve	FA-M (b) vs. ECR	Absolute power (2–30 Hz) without a priori definition of frequency bands, 1/f slope, IAP, IAF *19 electrodes	-	-	-	-	-	N/A	n.s. for all 4 outcomes (power change across whole frequency spectrum, IAP, IAF, 1/f slope)
	Other	long-term (varied) vs. naïve	FA-M (b) vs. ECR	Absolute power (2–30 Hz) without a priori definition of frequency bands, 1/f slope, IAP, IAF *19 electrodes	-	-	-	-	-	N/A	Decreased global power in alpha/beta range (9–30 Hz); n.s. differences for change in IAF and IAP; steeper 1/f slope.
	Other	long-term (varied)	FA-M (b) vs. MW - *experience sampling*	Absolute power (2–30 Hz) without a priori definition of frequency bands, 1/f slope, IAP, IAF *19 electrodes	-	-	-	-	-	N/A	n.s. for all 4 outcomes (power change across whole frequency spectrum, IAP, IAF, 1/f slope)
	Other	naïve	FA-M (b) vs. MW - *experience sampling*	Absolute power (2–30 Hz) without a priori definition of frequency bands, 1/f slope, IAP, IAF *19 electrodes	-	-	-	-	-	N/A	Decreased global power in alpha/beta range (10.5–25 Hz); decreased IAP (frontal).
	Other	long-term (varied) vs. naïve	FA-M (b) vs. MW - *experience sampling*	Absolute power (2–30 Hz) without a priori definition of frequency bands, 1/f slope, IAP, IAF *19 electrodes	-	-	-	-	-	N/A	Increased global power in theta and alpha/beta ranges (3–25 Hz).
Kakumanu et al. (2018)	Within (rest)	novice (V), long-term ( *sen* , *tea* ) (V)	FA-M (b) vs. ECR	Absolute power for Gamma, Beta, Alpha, Theta, Delta *129 electrodes	ns	↑	↑	↑	↑	Beta, Alpha, Theta, Delta: global	
	Between	long-term ( *sen* & *tea* ) (V) vs. novice (V)	FA-M (b)	Absolute power for Gamma, Beta, Alpha, Theta, Delta *129 electrodes	↓	ns	↑	ns	ns	Gamma, Alpha: global	
Milz et al. (2014)	Within (rest)	naïve	FA-M (bc) vs. ECR	Absolute power for Gamma, Beta, Alpha, Theta, Delta *58 electrodes	ns	ns	↑	ns	ns	Alpha: F1.	
	Coherence/connectivity		FA-M (bc) vs. ECR	sLORETA (intracortical lagged) coherence for Gamma, Beta, Alpha, Theta, Delta *19 ROIs	ns	↓	↓	↓	ns	Beta: left MFG-right PCG; Alpha: within right MFG; Theta: left MFG-right IPL, right MFG-right cuneus.	
	Coherence/connectivity		FA-M (bc) vs. ECR	sLORETA (head-surface conventional) coherence for Gamma, Beta, Alpha, Theta, Delta *19 ROIs	↑	↑	ns	ns	ns	Gamma: within left anterior region; Beta: over right side, frontal midline to left and right side.	
Lavallee et al. (2011)	Within (rest)	novice (NR)	FA-M (b) vs. ECR	Absolute power for Gamma, Beta, Alpha, Theta, Delta *19 electrodes	↓	↑	ns	ns	ns	Gamma: right IPL; Beta: right ACC, left precuneus.	
	Between	novice (NR) vs. naïve - *controlling for baseline differences*	FA-M (b), relaxation	Absolute power for Gamma, Beta, Alpha, Theta, Delta *19 electrodes	ns	↑	ns	ns	ns	Beta: right PHG, fusiform gyrus, right ITG, and right MTG.	
Baijal & Srinivasan (2010)	Between	long-term (SS) vs. naïve	FA-M (b/m), relaxation	Absolute and relative power for Beta, Alpha, Theta, Delta *16 ROIs	-	ns	^7^ ↓	↑↓ ^8^	ns	Alpha: global; Theta (increase): right frontal-central, left frontal-central, and right frontal; Theta (decrease): left posterior-occipital, left parietal.	
Takahashi et al. (2005) ^2^	Within (control)	naïve	FA-M (bc) vs. PB	Absolute power for Beta, Alpha, Theta *6 electrodes	-	ns	↑	↑	-	Alpha: F3, F4, C3, C4; Theta: F3, F4.	
Murata et al. (2004)	Coherence/connectivity	naïve	FA-M (bc) vs. PB	Coherence for Beta, Alpha, Theta *4 electrode pairs	-	ns	↑	ns	-	Alpha: F3–F4 electrode pair.	
Dunn et al. (1999)	Within (rest)	novice (NR)	FA-M (b) vs. ECR	Absolute power for Beta, Alpha, Theta, Delta *19 electrodes	-	↑↓	↑	↓	↓	Beta: mix of regions; Alpha: posterior only; Theta: global; Delta: global.	
	Within (meditation)		FA-M (b) vs. OM	Absolute power for Beta, Alpha, Theta, Delta *19 electrodes	-	↑↓	↓	↓	↓	Beta: mix of regions; Alpha: central and posterior; Theta: frontal only; Delta: mix of regions.	
Duda et al. (2023)	Other	naïve	FA-M (bc) vs. ECR	PCA components corresponding to Delta-Theta-Alpha, Low Alpha, High Alpha, Alpha-Beta	-	-	-	-	-	N/A	Decreased amplitude of low (Pz, O1, O2) and high alpha (P3, Pz, P4, O1, O2) components; decreased amplitude of alpha-beta (Cz, Pz, P4, O1, O2) component.
	Other	naïve: post 30 days FA-M practice (T2) vs. baseline (T1)	FA-M (bc) vs. ECR	PCA components corresponding to Delta-Theta-Alpha, Low Alpha, High Alpha, Alpha-Beta	-	-	-	-	-	N/A	Decreased amplitude of delta-theta-alpha (Fz, Cz, Pz) and alpha-beta (Cz, Pz, P4, O1, O2) components with a smaller relative reduction at T2 vs. T1.
Pal et al. (2022)	Within (rest)	long-term (Dh)	FA-M (m) vs. ECR	Absolute and relative energy for Beta, Alpha, Theta, Delta *4 electrodes	-	ns	ns	↑	ns	Theta: Pz-A2 electrodes.	
Hagerty et al. (2013)	Within (rest)	long-term (Jh)*	FA-M (Jh) ^4^ vs. rest	Relative power for Gamma, Beta, Alpha, Theta *11 ROIs	↑	ns	↑	ns	-	Gamma: ACC, medial OFC; Alpha: global.	Difference in power between high-frequency bands (gamma & beta) minus the power in low-frequency bands (alpha & theta) showed increased activity for ACC and medial OFC, decreased activity for visual, auditory, Broca, orientation, and somatosensory regions.
Colgan et al. (2019)	Within (control)	novice (V)*	FA-M (b) vs. IMW ^5^	Absolute power for Gamma, Beta, Alpha, Theta *average across 18 electrodes	↑	↑	↑	↑	-	N/A	
Young et al. (2021)	Within (control)	long-term (Sha)	FA-M (b) vs. IMW	Absolute power for Beta, Alpha, Theta, Delta *16 electrodes	-	↓	ns	↓	ns	Beta: C4, O1; Theta: O1	
Yordanova et al. (2021) ^2^	Coherence/connectivity	long-term (TVB)	FA-M (b) vs. ECR	Imaginary part of coherence for Beta, Alpha, Theta *fronto-parietal and medial frontal networks only	-	↑	↑	↑	-	Fronto-parietal network Beta & Alpha: right frontal-left parietal; Theta: within left frontoparietal. Medial frontal network Beta: within right medial frontal;Theta: medial frontal-left parietal.	Meditation experience was positively associated with: (i) right frontal-left parietal synchronization for beta and alpha bands, and (ii) medial frontal-left parietal synchronization for theta band.
Irrmischer et al. (2018) , **Study 1**	Within (rest)	novice (Zen, V) & naïve	FA-M (b) vs. ECR	Absolute power for Gamma, Beta, Alpha, Theta, Delta *32 electrodes	ns	ns	ns	ns	ns	N/A	
Irrmischer et al. (2018) , **Study 2**	Within (rest)	novice (varied) & naïve	FA-M (b) vs. ECR	Absolute power for Gamma, Beta, Alpha, Theta, Delta *32 electrodes	ns	ns	↑	ns	ns	Alpha: frontal, central, parietal, and occipital.	Decrease in alpha frequency oscillations in frontal and parietal regions.
Amihai & Kozhevnikov (2014)	Within (rest)	long-term (Vaj)	FA-M (IDI) vs. ECR	Absolute power for Gamma, Beta, Alpha, Theta, Delta *9 electrodes grouped into left, right, and center regions	↓	↓	ns	ns	↓	Gamma: left, right, and center; Beta & Delta: average across regions.	
	Within (meditation)		FA-M (IDI) vs. OM	Absolute power for Gamma, Beta, Alpha, Theta, Delta *9 electrodes grouped into left, right, and center regions	ns	ns	ns	ns	ns	N/A	
	Coherence/connectivity		FA-M (IDI) vs. ECR	Coherence for Gamma, Beta, Alpha *Frontal, Fronto-Central, Posterior, Fronto-Posterior	ns	↑	↓	-	-	Beta: fronto-central; Alpha: frontal.	
	Within (rest)	long-term (TVB)	FA-M (ko) vs. ECR	Absolute power for Gamma, Beta, Alpha, Theta, Delta *9 electrodes grouped into left, right, and center regions	ns	ns	↓	ns	ns	Alpha: left, right, and center.	
	Within (meditation)		FA-M (ko) vs. OM	Absolute power for Gamma, Beta, Alpha, Theta, Delta *9 electrodes grouped into left, right, and center regions	ns	ns	ns	ns	ns	N/A	
	Coherence/connectivity		FA-M (ko) vs. ECR	Coherence for Gamma, Beta, Alpha *Frontal, Fronto-Central, Posterior, Fronto-Posterior	ns	ns	↓	-	-	Alpha: frontal.	
Sliwowski et al. (2023)	Within (control)	naïve	FA-M (b) vs. listening	Relative power for Gamma, Alpha, Theta *frontal, central, and parietal-occipital regions	ns	-	ns	ns	-	N/A	
	Within (rest)		FA-M (b) vs. ECR	Relative power for Gamma, Alpha, Theta *frontal, central, and parietal-occipital regions	ns	-	↓	ns	-	Alpha: central.	Decreased theta hemispheric power asymmetry at parietal-occipital region.
	Coherence/connectivity		FA-M (b) vs. listening	Activity outflow for Gamma, Alpha, Theta *frontal, central, and parietal-occipital regions	ns	-	↓	↓	-	Alpha: frontal, central, and parietal-occipital; Theta: central and parietal-occipital.	
	Coherence/connectivity		FA-M (b) vs. ECR	Activity outflow for Gamma, Alpha, Theta *frontal, central, and parietal-occipital regions	ns	-	↓	↓	-	Alpha, Theta: frontal and central.	
Thomas et al. (2014)	Other	long-term (SY) vs. novice (SY)	FA-M (b)	CSD using eLORETA for Gamma, Beta, Alpha, Theta, Delta *25 electrodes	↑	↑	↓	↓	ns	Gamma: fusiform gyrus; Beta: sub-gyral temporal lobe; Alpha: precentral gyrus, IPL; Theta: IPL.	
	Other		FA-M (m)	CSD using eLORETA for Gamma, Beta, Alpha, Theta, Delta *25 electrodes	↑	↑	↓	↓	ns	Gamma: insula; Beta: rectal gyrus; Alpha: precentral gyrus; Theta: IPL.	
Braboszcz & Delorme (2011)	Within (MW)	not stated	FA-M (bc) vs. MW - *experience sampling*	Absolute power for Beta, Alpha, Theta, Delta *128 electrodes	-	↑	↑	↓	↓	Beta: fronto-lateral; Alpha: occipital; Theta, Delta: global.	
Rodriguez-Larios et al., (2024)	Other	novice (MBSR)	FA-M (b) vs. MW *- experience sampling*	Oscillatory burst coverage ^9^ (1–30 Hz) without a priori definition of frequency bands, IAF *64 electrodes	-	-	-	-	-	N/A	Decreased posterior theta/delta (~3–6 Hz) oscillatory burst coverage.Faster peak frequency of theta oscillations.IAF: n.s.
	Other		FA-M (b) vs. drowsiness *- experience sampling*	Oscillatory burst coverage ^9^ (1–30 Hz) without a priori definition of frequency bands, IAF *64 electrodes	-	-	-	-	-	N/A	Decreased global theta/delta (~3–5 Hz) oscillatory burst coverage.Faster peak frequency of theta oscillations.IAF: n.s.
	Other	novice (MBSR) vs. naïve (waitlist): post-training (T2) vs. baseline (T1)	FA-M (b) *- experience sampling*	Oscillatory burst coverage ^9^ (1–30 Hz) without a priori definition of frequency bands, IAF *64 electrodes	-	-	-	-	-	N/A	n.s. for either burst coverage across whole frequency range or IAF.
	Other	novice (MBSR) vs. naïve (waitlist): post-training (T2) vs. baseline (T1)	FA-M (b)	Oscillatory burst coverage ^9^ (1–30 Hz) without a priori definition of frequency bands, IAF *64 electrodes	-	-	-	-	-	N/A	n.s. for burst coverage across whole frequency range.IAF: frontal.
Heinilä et al. (2024)	Within (control)	mixed (NR)	FA-M (b) vs. IMW (future planning)	Absolute power for Beta, Alpha *306 channels (MEG)	-	↑↓	↓	-	-	Beta: right parietal, left temporal; Alpha: right parietal.	
	Within (control)		FA-M (b) vs. IMW (anxious thinking)	Absolute power for Beta, Alpha *306 channels (MEG)	-	↑↓	↑	-	-	Beta: right parietal, left temporal; Alpha: left temporal.	
Sihn et al. (2024)	Other	long-term (HY) vs. naïve	FA-M (b/m), FA-M (b)	Amplitude of SCPs, phase amplitude coupling for alpha-SCP and gamma-SCP. *64 electrodes	-	-	-	-	-	N/A	n.s. for SCP amplitude.Decreased alpha-SCP phase amplitude coupling (3/64 electrodes), n.s. differences for gamma-SCP.
Neri et al. (2024)	Other	mixed (TB)	FA-M (NR) vs. analytical meditation	Changes in absolute power for Gamma, Beta, Alpha, Theta, Delta, and Alpha peak ^10^ compared with baseline. *19 electrodes	-	-	-	-	-	N/A	Greater positive changes for beta, alpha, theta, and delta bandwidths, and alpha peak values.Greater negative changes for the alpha bandwidth.

Note: A comprehensive overview of all spectral analysis outcomes by study. *Indicates case studies.^1^Alpha-Theta state was characterized either by (i) >50% alpha waves or (ii) a predominance of theta activity on a low voltage mixed background.^2^Secondary analyses using datasets whose original manuscripts are also included in the review.^3^Global field power was calculated by averaging power over all electrodes and frequency bands.^4^The Jhanas are states of deep concentrative absorption arising from meditation.^5^Separate FA-M versus IMW block comparisons were conducted (i.e., FA-M 1 vs. IMW 1, FA-M 1 vs. IMW 2, FA-M 2 vs. IMW 1). Significant findings were either observed across all comparisons (alpha) or two out of three comparisons (gamma, beta, theta).^6^Significant finding was for relative power only, although there was a trending result for absolute power.^7^Significant finding was for absolute power only.^8^Significant findings were for relative power only.^9^Oscillatory burst coverage represents the quantity of time in which oscillatory activity (exceeding aperiodic activity) was detected during trials.^10^Alpha peak corresponds to the maximum positive variation of the height of the alpha peak across the entire condition. Participant group(s) abbreviations: TB = Tibetan Buddhism, HY = Himalayan Yoga, IS = Isha Shoonya, V = Vipassana, AM = Ananda Marga, Ana = Anapanasati, TVB = Theravada Buddhism, Sha = Shamatha, sen = senior practitioners, tea = meditation teachers, SS = Sahaj Samadhi, Dh = Dharana, Jh = Jhana, Vaj = Vajrayana, SY = Satyananda Yoga. MBSR = mindfulness-based stress reduction, NR = Not Reported. Condition(s) abbreviations: FA-M = focused attention meditation, fc = fixation cross, OM = open monitoring meditation, b = breath, m = mantra, IMW = instructed mind wandering, ECR = eyes-closed rest, bc = breath counting, PB = paced breathing, MW = mind wandering, EM = emptiness meditation, fh = fore-head, PM = presence meditation, NR = not reported, IDI = internal deity image, ko = kasina object. Other abbreviations: G = gamma, B = beta, A = alpha, T = theta, D = delta, ROIs = regions of interest, ns = not significant, N/A = not applicable, SCP = slow cortical potentials, MEG = magnetoencephalography, sLORETA = standardized low resolution brain electromagnetic tomography, PCA = principal component analysis, CSD = current source density, eLORETA = exact low resolution brain electromagnetic tomography, IAF = individual alpha frequency, IAP = individual alpha power, IPL = inferior parietal lobe.

##### 3.4.1.1 Delta frequency

There were 18 studies that examined the delta frequency band, across which a total of 28 within- and between-group comparisons were conducted, fewer than any other frequency band. Specifically, there were 23 comparisons of delta spectral power and 5 comparisons of functional connectivity measures.

Among the studies focusing on delta spectral power, 20 within-group comparisons were conducted, where FA was compared with a resting state (n = 10), a control condition (n = 1), a different meditation technique (n = 6), or naturally occurring mind wandering as part of an experience-sampling paradigm (n = 2). Additionally, one longitudinal study compared FA across different timepoints during a 3-month retreat (Saggar et al., 2012). The findings revealed only one instance of increased delta power for FA compared with rest (Kakumanu et al., 2018). In contrast, six instances of decreased delta power were reported for FA compared with rest (n = 3) (Amihai & Kozhevnikov, 2014;Dunn et al., 1999;Walter & Hinterberger, 2022), an OM meditation condition (n = 1) (Dunn et al., 1999), or mind wandering (n = 2) (Braboszcz & Delorme, 2011;van Son et al., 2019). There were also 13 within-group condition comparisons reporting non-significant findings for FA compared with rest (n = 6) (Amihai & Kozhevnikov, 2014;Irrmischer et al., 2018;Lavallee et al., 2011;Milz et al., 2014;Pal et al., 2022), a control condition (n = 1) (Young et al., 2021), a different meditation technique (n = 5) (Amihai & Kozhevnikov, 2014;Hinterberger et al., 2014;Walter & Hinterberger, 2022), and in a longitudinal comparison during a 3-month retreat (mid- and post-retreat vs. pre-retreat baseline) (Saggar et al., 2012). For between-group comparisons, three studies investigated delta power differences for long-term versus novice meditators (n = 1), long-term meditators versus meditation-naïve participants (n = 1), or novice meditators versus meditation-naïve participants (n = 1) during FA, all of which yielded non-significant results (Baijal & Srinivasan, 2010;Kakumanu et al., 2018;Lavallee et al., 2011).

There were five within-group comparisons examining functional connectivity measures in the delta band, comparing FA with rest (n = 4) or OM meditation (n = 1). While one comparison yielded increased delta coherence for FA compared with rest (Yordanova et al., 2020), the remaining four comparisons reported non-significant findings (Marzetti et al., 2014;Milz et al., 2014).

##### 3.4.1.2 Theta frequency

There were 31 studies that examined the theta frequency band, resulting in a total of 46 within- and between-group comparisons. Specifically, there were 37 comparisons of theta spectral power and nine comparisons of functional connectivity measures.

Among the studies that investigated theta spectral power, there were 33 within-group comparisons, where FA was compared with a resting state (n = 13), a control condition (n = 7), a different meditation technique (n = 7), naturally occurring mind wandering (n = 5), or different timepoints in a longitudinal study (n = 1). The results indicated seven instances of increased theta power during FA relative to rest (n = 3) (Kakumanu et al., 2018;Pal et al., 2022;Tsai et al., 2013), a control condition (n = 3) (Colgan et al., 2019;Kubota et al., 2001;Takahashi et al., 2005), or mind wandering (n = 1) (Brandmeyer & Delorme, 2018). Conversely, nine instances of decreased theta power were observed during FA compared with rest (n = 2) (Dunn et al., 1999;Walter & Hinterberger, 2022), a control condition (n = 2) (Park & Park, 2012;Young et al., 2021), other meditation techniques (n = 2) (Dunn et al., 1999;Tsai et al., 2013), or mind wandering (n = 3) (Braboszcz & Delorme, 2011;Rodriguez-Larios & Alaerts, 2021;van Son et al., 2019). Additionally, 17 within-group comparisons yielded non-significant results for FA compared with rest (n = 8) (Amihai & Kozhevnikov, 2014;Hagerty et al., 2013;Irrmischer et al., 2018;Lavallee et al., 2011;Milz et al., 2014;Śliwowski et al., 2023), a control condition (n = 2) (Park, 2021;Śliwowski et al., 2023), other meditation techniques (n = 5) (Amihai & Kozhevnikov, 2014;Hinterberger et al., 2014;Walter & Hinterberger, 2022), mind wandering (n = 1) (Brandmeyer & Delorme, 2018), and in a longitudinal comparison (n = 1) (Saggar et al., 2012). There were also four between-group comparisons of theta spectral power which compared long-term meditators versus either novice meditators (n = 1) or meditation-naïve participants (n = 2) and novice meditators versus meditation-naïve participants (n = 1) during FA. Among these, one comparison yielded increased theta power (Corby et al., 1978), and one yielded mixed evidence (Baijal & Srinivasan, 2010) for long-term meditators compared with meditation-naive participants. The remaining two comparisons showed non-significant differences (Kakumanu et al., 2018;Lavallee et al., 2011).

There were nine within-group comparisons examining functional connectivity measures in the theta band, comparing FA with rest (n = 6), a control condition (n = 2), or OM meditation (n = 1). Increased theta coherence was found in two instances, both compared with rest (Yordanova et al., 2020,^2021^). In contrast, three instances of decreased theta coherence were reported during FA compared with rest (n = 2) and a passive listening control task (n = 1) (Milz et al., 2014;Śliwowski et al., 2023). The remaining four comparisons observed non-significant findings for theta functional connectivity (Marzetti et al., 2014;Milz et al., 2014;Murata et al., 2004).

##### 3.4.1.3 Alpha frequency

There were 35 studies that investigated the alpha frequency band, resulting in a total of 58 within- and between-group comparisons, more than any other frequency band. Specifically, there were 47 comparisons of alpha spectral power and 11 comparisons of functional connectivity measures.

Within the realm of alpha spectral power, 40 within-group comparisons were conducted, where FA was compared with rest (n = 14), a control condition (n = 11), other meditation techniques (n = 9), naturally occurring mind wandering (n = 5), or different timepoints in a longitudinal study (n = 1). The findings revealed 16 instances of increased alpha power during FA compared with rest (n = 6) (Chotipanich et al., 2021;Dunn et al., 1999;Hagerty et al., 2013;Irrmischer et al., 2018;Kakumanu et al., 2018;Milz et al., 2014), a control condition (n = 6) (Colgan et al., 2019;Fucci et al., 2018,^2022^;Heinilä et al., 2024;Park & Park, 2012;Takahashi et al., 2005), or mind wandering (n = 4) (Braboszcz & Delorme, 2011;Brandmeyer & Delorme, 2018;Rodriguez-Larios & Alaerts, 2021;van Son et al., 2019). In contrast, there were eight instances where alpha power decreased in comparison with rest (n = 2) (Amihai & Kozhevnikov, 2014;Śliwowski et al., 2023), a control condition (n = 1) (Heinilä et al., 2024), or other meditation techniques (n = 5) (Dunn et al., 1999;Fucci et al., 2018,^2022^;Tsai et al., 2013;Walter & Hinterberger, 2022). Additionally, 16 within-group comparisons reported non-significant findings for FA compared with rest (n = 6) (Amihai & Kozhevnikov, 2014;Irrmischer et al., 2018;Lavallee et al., 2011;Pal et al., 2022;Tsai et al., 2013;Walter & Hinterberger, 2022), a control condition (n = 4) (Braboszcz et al., 2017;Park, 2021;Śliwowski et al., 2023;Young et al., 2021), other meditation techniques (n = 4) (Amihai & Kozhevnikov, 2014;Hinterberger et al., 2014;Walter & Hinterberger, 2022), mind wandering (n = 1) (Brandmeyer & Delorme, 2018), and in the longitudinal study (n = 1) (Saggar et al., 2012). Seven between-group comparisons of alpha spectral power were also conducted, where long-term meditators were compared with novices (n = 1), meditation-naïve participants (n = 4), or another group of long-term meditators from a different tradition (n = 1) during FA. Additionally, there was one comparison of novice meditators versus meditation-naïve participants. Among these comparisons, two instances each of increased alpha power (Corby et al., 1978;Kakumanu et al., 2018) and decreased alpha power (Baijal & Srinivasan, 2010;Braboszcz et al., 2017) were reported. The remaining three comparisons yielded non-significant findings (Fucci et al., 2018,^2022^;Lavallee et al., 2011).

Regarding functional connectivity measures, 11 within-group comparisons were conducted, comparing FA with rest (n = 8), a control condition (n = 2), or OM meditation (n = 1). There were three instances of increased alpha coherence, compared with rest (n = 2) and paced breathing (n = 1) (Murata et al., 2004;Yordanova et al., 2020,^2021^). Conversely, seven instances of decreased alpha coherence/functional connectivity were observed compared with rest (n = 5), passive listening (n = 1), and OM meditation (n = 1) (Amihai & Kozhevnikov, 2014;Marzetti et al., 2014;Milz et al., 2014;Śliwowski et al., 2023). Only one comparison reported a non-significant result (Milz et al., 2014).

##### 3.4.1.4 Beta frequency

There were 26 studies that explored the beta frequency, resulting in a total of 39 within- and between-group comparisons. Specifically, there were 30 comparisons of beta spectral power and nine comparisons of functional connectivity measures.

In the domain of beta spectral power, 27 within-group comparisons were conducted, where FA was compared with resting states (n = 11), a control condition (n = 7), other meditation techniques (n = 6), mind wandering (n = 2), or different timepoints in a longitudinal study (n = 1). These findings included six instances of increased beta power during FA compared with rest (n = 2) (Kakumanu et al., 2018;Lavallee et al., 2011), a control condition (n = 2) (Colgan et al., 2019;Park & Park, 2012), or naturally occurring mind wandering (n = 2) (Braboszcz & Delorme, 2011;van Son et al., 2019). Conversely, three instances of decreased beta power were observed during FA compared with rest (Amihai & Kozhevnikov, 2014), instructed mind wandering (Young et al., 2021), or within the longitudinal study (Saggar et al., 2012). Notably, two studies reported mixed results for beta power across four within-group comparisons of FA (Dunn et al., 1999;Heinilä et al., 2024). Additionally, 14 within-group comparisons found non-significant differences in beta power during FA compared with rest (n = 7) (Amihai & Kozhevnikov, 2014;Hagerty et al., 2013;Irrmischer et al., 2018;Milz et al., 2014;Pal et al., 2022;Walter & Hinterberger, 2022), a control condition (n = 2) (Park, 2021;Takahashi et al., 2005), or other meditation techniques (n = 5) (Amihai & Kozhevnikov, 2014;Hinterberger et al., 2014;Walter & Hinterberger, 2022). Three between-group comparisons of beta spectral power were conducted, comparing long-term meditators with novices (n = 1) or meditation-naïve participants (n = 1) and comparing novices with meditation-naïve participants (n = 1) during FA. One comparison reported increased beta power for novice meditators compared with meditation-naïve participants (Lavallee et al., 2011), while the remaining two comparisons found non-significant results (Baijal & Srinivasan, 2010;Kakumanu et al., 2018).

There were nine within-group comparisons examining functional connectivity measures in the beta band, comparing FA with resting states (n = 7), a control condition (n = 1), or OM meditation (n = 1). There were four instances of increased beta coherence during FA, all compared with rest (Amihai & Kozhevnikov, 2014;Milz et al., 2014;Yordanova et al., 2020,^2021^), and one instance of decreased beta coherence, also compared with rest (Milz et al., 2014). The remaining four comparisons reported non-significant findings regarding beta functional connectivity during FA (Amihai & Kozhevnikov, 2014;Marzetti et al., 2014;Murata et al., 2004).

##### 3.4.1.5 Gamma frequency

There were 17 studies that investigated the gamma frequency band, resulting in a total of 35 within- and between-group comparisons. Specifically, there were 26 comparisons of gamma spectral power and 9 comparisons of functional connectivity measures.

In the context of gamma spectral power, 22 within-group comparisons were conducted, where FA was compared with resting states (n = 10), a control condition (n = 4), other meditation technique (n = 7), or different timepoints in a longitudinal study (n = 1). The findings included six instances of increased gamma power during FA compared with rest (n = 2) (Hagerty et al., 2013;Walter & Hinterberger, 2022), a control condition (n = 1) (Colgan et al., 2019), or other meditation techniques (n = 3) (Braboszcz et al., 2017;Hinterberger et al., 2014;Walter & Hinterberger, 2022). Conversely, three instances of decreased gamma power were observed during FA compared with rest (n = 2) (Amihai & Kozhevnikov, 2014;Lavallee et al., 2011) or a control reading task (n = 1) (Fucci et al., 2018). Additionally, 13 within-group comparisons reported non-significant differences in gamma power for FA compared with rest (n = 6) (Amihai & Kozhevnikov, 2014;Irrmischer et al., 2018;Kakumanu et al., 2018;Milz et al., 2014;Śliwowski et al., 2023), a control condition (n = 2) (Braboszcz et al., 2017;Śliwowski et al., 2023), other meditation techniques (n = 4) (Amihai & Kozhevnikov, 2014;Fucci et al., 2018;Walter & Hinterberger, 2022), or in the longitudinal study (Saggar et al., 2012). There were also four between-group comparisons of gamma spectral power, where long-term meditators were compared with novices (n = 2) or meditation-naïve participants (n = 1) and novices were compared with meditation-naïve participants (n = 1) during FA. These comparisons revealed two instances of increased gamma power (Braboszcz et al., 2017;Fucci et al., 2018) and one instance of decreased gamma power (Kakumanu et al., 2018) for the long-term meditator comparisons. The remaining between-group novice meditator comparison was non-significant (Lavallee et al., 2011).

Regarding functional connectivity measures in the gamma band, eight of the nine reported results were within-group condition comparisons, where FA was compared with resting conditions (n = 6), a passive listening control condition (n = 1), or OM meditation (n = 1). Among these comparisons, only one yielded significant findings, indicating increased gamma coherence during FA compared with rest (Milz et al., 2014). The other comparisons reported non-significant findings (Amihai & Kozhevnikov, 2014;Marzetti et al., 2014;Milz et al., 2014;Śliwowski et al., 2023). The one between-group comparison found increased gamma coherence during FA for long-term Himalayan Yoga practitioners compared with meditation-naïve participants (Martínez Vivot et al., 2020).

##### 3.4.1.6 Unique spectral outcomes

Accompanying the spectral analysis results summarized thus far, there were also nine studies that reported unique outcomes related to spectral analysis. Here, we provide a brief overview of each study, while a complete list of significant findings can be found inTable 3under the comparison type “Other.”

With long-term meditators and meditation-naïve participants,Rodriguez-Larios et al. (2021)conducted both within- and between-group comparisons regarding four EEG outcome measures—absolute power without any a priori definition of the frequency bands, 1/f slope, individual alpha power (IAP), and individual alpha frequency (IAF)—for FA compared with both a resting-state condition and naturally occurring mind wandering during an experience sampling paradigm. Also using an experience-sampling paradigm,Rodriguez-Larios et al. (2024)conducted within-group comparisons of novice meditators regarding IAF and oscillatory burst coverage for FA compared with self-reported states of mind wandering and drowsiness. Additionally, in the same study, between-group comparisons were conducted to evaluate changes in these outcomes during both interrupted (i.e., experience sampling) and uninterrupted FA periods following 8 weeks of either mindfulness-based stress reduction (MBSR) training or a waitlist control. Among a group of Tibetan Buddhist meditators with mixed levels of meditation experience,Neri et al. (2024)assessed both positive and negative deviations (relative to a within-condition baseline) in alpha peak values and spectral power across all frequency bandwidths for FA compared with analytical meditation.Duda et al. (2023)utilized frequency Principal Component Analyses (PCA) to investigate changes in spectral components across multi-frequency band components (e.g., Delta-Theta-Alpha) during FA compared with rest among meditation-naïve participants at baseline and after 1 month of daily practice.Thomas et al. (2014)used eLORETA to compare differences in cortical source activity during two types of FA—breath and mantra—between Satyananda Yoga students and teachers. In an older study,Elson et al. (1977)compared the proportion of time spent in an “Alpha-Theta” state between long-term Ananda Marga meditators performing FA and meditation-naïve participants instructed to remain wakefully relaxed. Two studies assessed spectral power outcomes pertaining to slow cortical potentials (SCPs), low frequency oscillatory signals (<0.1 Hz) that are less commonly examined than the conventional frequency bandwidths previously discussed. Specifically,Sihn et al. (2024)assessed differences in the amplitude of SCPs and its phase amplitude coupling with the alpha and gamma bandwidths for long-term Himalayan Yoga practitioners compared with meditation-naïve participants. During FA,Jo et al. (2019)investigated within-SCP phase synchrony for long-term meditators and meditation-naïve participants before and after completing an 8-week MBSR program. Finally, among meditators with varying backgrounds and experience levels,López et al. (2022)found that the subfactor “mental quieting” of a newly proposed Meditative State Scale (MSS) was positively correlated with increased gamma power across several brain regions during FA.

#### 3.4.2 Non-linear measures

As observed thus far, most studies investigating the neurophysiological mechanisms associated with FA have focused on linear EEG measures, primarily spectral power. However, the brain operates as a complex, chaotic system characterized by non-linear dynamics (Breakspear, 2017;Chialvo, 2010;McKenna et al., 1994;Sporns, 2022). In this review, seven studies examined non-linear EEG measures during FA (D’Andrea et al., 2024;Irrmischer et al., 2018;Kakumanu et al., 2018;Lu & Rodriguez-Larios, 2022;Martínez Vivot et al., 2020;Ventura et al., 2024;Walter & Hinterberger, 2022), which can be grouped under the frameworks of (i) complexity, which includes multiscale entropy (MSE), sample entropy (SE), permutation entropy (PE), fractal dimension (HFD), and Lempel-Ziv complexity (LZC), and (ii) criticality, which includes the critical scaling exponent (SNZ) and long-range temporal correlations (LRTC).

Collectively, these seven studies observed several significant within- and between-group differences in complexity and criticality measures during FA. For long-term meditators, FA was linked to greater neural complexity (MSE) and reduced criticality (SNZ, LRTC) than rest (Walter & Hinterberger, 2022). In the same study, FA also demonstrated greater neural complexity (MSE) and reduced criticality (SNZ) when compared with “emptiness” and “presence” meditation techniques. A similar pattern was observed byD’Andrea et al. (2024)when investigating the complexity and criticality of dynamic transitions between MEG microstates among a group of Theravada Buddhist monks. Specifically, FA was associated with greater complexity (LZC) and decreased criticality (LRTC) than rest. In this case, however, FA was associated with lower complexity than OM meditation, an effect that was positively associated with meditation proficiency. In another study, long-term Himalayan Yoga practitioners showed greater neural complexity (SE) in the high gamma band during FA than meditation-naïve participants (Martínez Vivot et al., 2020). During an experience sampling paradigm, meditation-naïve participants performing FA exhibited increased neural complexity (HFD, SE, LZC) during states of breath focus compared with mind wandering (Lu & Rodriguez-Larios, 2022).

There were also a few studies that only examined criticality measures. Across two independent studies,Irrmischer et al. (2018)showed that FA was associated with reduced criticality (LRTC) compared with rest for long-term meditators but not meditation-naïve participants. This effect was observed across multiple frequency bands with widespread topography, indicating a global change in brain state. Furthermore, the subjective and training-related effects of FA on criticality were explored in one of the studies, where the ability to suppress LRTC during FA was associated with greater self-reported absorption experience and increased after 1 year of additional training. Across several within- and between-group comparisons,Ventura et al. (2024)robustly demonstrated that FA is associated with a decreased duration of the autocorrelation window (ACW)—which, as with LRTC, measures the correlation of neural activity with itself over time—compared with practices requiring a wider attentional focus, such as OM meditation.

Finally, among three participant groups of varying proficiencies, only meditation teachers—the most proficient group—showed increased neural complexity (PE) during FA compared with rest (Kakumanu et al., 2018). However, when comparing between groups, novice meditators exhibited greater neural complexity (HFD, PE) during FA than long-term meditators and meditation teachers. This between-group finding represents the only counter-evidence to the general trend observed in this review, which showed increased complexity and decreased criticality measures associated with FA compared with control conditions and other meditation techniques, or as a function of participants’ level of meditation proficiency.

#### 3.4.3 Event-related potentials (ERPs)

There were eight studies that examined ERP components during FA (Atchley et al., 2016;Biedermann et al., 2016;Braboszcz & Delorme, 2011;Fucci et al., 2018,^2022^;Lin, White, Viravan, et al., 2024;Lin, White, Wu, et al., 2024;Telles et al., 2015). Most of these studies focused on components of auditory evoked potentials (AEPs), examining the neural responses to auditory stimuli during FA.Telles et al. (2015)investigated the peak latency and amplitude of multiple AEP components—P1, N1, P2, and N2—among novice meditators. A decrease in the peak latency of the P2 component was observed during FA compared with rest, representing the only statistically significant finding. InAtchley et al. (2016), reduced N2 and P3 amplitudes across midline electrodes were observed during FA—specifically, a breath counting task—compared with a tone identification control condition for meditation-naïve participants, novice meditators, and long-term meditators. Additionally, an effect of meditation experience was observed, where the two meditator groups displayed greater changes in peak-to-trough P3 amplitudes in accordance with the experimental condition (i.e., lower amplitudes during breath counting, higher amplitudes during the control condition) compared with the meditation-naïve group. In addition to these components, other studies have also examined other well-characterized components of the AEP, namely, the Mismatch Negativity (MMN) and Late Frontal Negativity (LFN), yielding mixed results.

InFucci et al. (2018), MMN and LFN amplitudes were investigated in frontal brain regions. MMN amplitude increased during FA compared with rest for long-term meditators but not meditation-naïve participants. Relatedly, a significantly higher MMN amplitude was observed for long-term meditators compared with meditation-naïve participants during FA. Across both participant groups, LFN amplitude increased during FA compared with rest. When including both components in an integrated statistical model, long-term meditators showed (i) increased MMN and decreased LFN amplitude for FA compared with “open presence” meditation and (ii) increased MMN and LFN amplitude for FA compared with rest. However, in a replication attempt of the MMN finding by the same group with a larger sample size, there was no change in amplitude during FA compared with a control condition among either long-term meditators or meditation-naïve participants (Fucci et al., 2022).Biedermann et al. (2016)investigated N1, P2, and MMN components among long-term meditators and meditation-naïve participants during FA compared with a non-meditation control condition. There were no significant changes in MMN amplitude during FA compared with the control condition for either participant group. Although long-term meditators exhibited a greater MMN amplitude than the meditation-naïve participants during FA, this trend was non-significant. Hence, there were no significant state or state by trait interaction effects for the MMN in this study. However, a significant decrease in N1 amplitude and increase in P2 amplitude were observed during FA compared with a non-meditation control condition in the meditation-naïve group but not long-term meditators. Finally, during an experience-sampling paradigm, meditation-naïve participants displayed decreased P2 amplitude over fronto-central regions and increased MMN amplitude over the right frontal region during states of breath focus compared with mind wandering (Braboszcz & Delorme, 2011).

While most studies investigated AEPs, two studies examined components of visually evoked ERPs using a single dataset of meditation-naïve participants who completed cognitive (i.e., Flanker) and emotion provocation (i.e., picture viewing) tasks while maintaining an FA or OM state (Lin, White, Viravan, et al., 2024;Lin, White, Wu, et al., 2024). During the Flanker task, FA was not found to exert any effect on either the stimulus-locked P3 or the response-locked error-related negativity (ERN) and error positivity (Pe) components (Lin, White, Viravan, et al., 2024). Conversely, during the picture viewing task, higher negative arousal ratings led to an observed dissociation between the FA and OM states with respect to the late positive potential (LPP), a well-established neural marker of emotion processing. Specifically, FA and OM were associated with either enhanced or reduced LPP response, respectively (Lin, White, Wu, et al., 2024).

#### 3.4.4 Classification methods

There were six studies that applied machine-learning classification methods to neurophysiological data. Among these studies, classification methods were used to differentiate between long-term meditators and meditation-naïve participants (n = 2), to distinguish between FA and OM states (n = 2), to classify meditators based on years of experience (n = 1), or to detect mind wandering episodes during FA (n = 1) (Calvetti et al., 2021;Dadhich et al., 2024;Kopal et al., 2014;Y.-H. Lee et al., 2017;Martínez Vivot et al., 2020;Pandey et al., 2022).

Martínez Vivot et al. (2020)employed multivariate random forest classifiers to distinguish between Himalayan Yoga practitioners and meditation-naïve participants during FA with high accuracy in the low gamma band. Using the same dataset,Pandey et al. (2022)replicated this finding with a different classification approach, specifically binary classification via support vector machine, achieving a maximum classification accuracy of 84.8% using a low gamma band feature.Calvetti et al. (2021)utilized MEG data from two Buddhist monks and applied linear discriminant analysis to clearly separate brain activity during different states (i.e., FA, OM, rest). They identified several brain regions contributing to this separation, including the cingulate cortex, insular cortex, nucleus accumbens, caudate and putamen nuclei, thalamus, and amygdala.Kopal et al. (2014)successfully differentiated between FA and OM states among both long-term meditators and meditation-naïve participants by identifying an optimal threshold of complex continuous wavelet coherence (WTC) measures.Lee et al. (2017)employed two machine-based classifiers—an artificial neural network and a support vector machine—to classify meditators according to their years of experience in 10-year intervals. Both classifiers achieved high accuracy rates exceeding 98%. Finally,Dadhich et al. (2024)used the flexible analytic wavelet transform to decompose raw EEG data into more representative sub-bands. This approach yielded high classification accuracy (~92.5%), sensitivity (~93.5%), and specificity (~92%) for detecting mind wandering episodes during FA.

#### 3.4.5 Consumer-grade EEG

There were four studies reporting outcomes using consumer-grade EEG devices, specifically the Muse (n = 3) and Emotiv EPOC (n = 1) (Hunkin et al., 2021;Lazarou et al., 2023;Sharma et al., 2022;Steinhubl et al., 2015). In one study, states of “mind wandering,” as defined by the Muse based on brain activity, were linked to task-based measures of focused attention, such as breath counting errors (Hunkin et al., 2021). Additionally, Muse device metrics during home practice explained approximately 30% of the variance in self-reported trait mindfulness, attentional control, non-attachment, and decentering. In a separate study, the Muse was used to quantify the time participants spent in a “calm state” while comparing three different FA techniques: mantra, breath, and external-point meditation (Sharma et al., 2022). Results indicated that FA techniques with an internal focus (mantra and breath) were superior to external-point meditation in terms of time spent in the calm state. Another study employed the Muse device to investigate changes in brain rhythms during two FA techniques (breath and mantra) among individuals with subjective cognitive decline and mild cognitive impairment, and healthy controls in a simulated home environment (Lazarou et al., 2023). The findings revealed significant changes in brain rhythms, predominantly in the beta and theta bands, which were largely consistent across all participant groups. Using the Emotiv EPOC,Steinhubl et al. (2015)found that both novice meditators and meditation-naïve participants showed significant increases in their “meditation score” and relative gamma power during FA compared with rest. Notably, the degree of change in both outcomes was greater for novice meditators than for meditation-naïve participants. However, after completing a week-long yoga and meditation retreat, this between-group difference was no longer statistically significant.

#### 3.4.6 Hyperscanning

There were two studies that implemented hyperscanning during FA (Coomans et al., 2021;Matiz et al., 2021). In the study byCoomans et al. (2021), inter-subject EEG coherence was measured in meditation-naïve dyads during individual and joint practice of guided FA. The findings indicated increased inter-subject alpha coherence in frontal and temporal brain regions during joint FA practice compared with individual practice, suggesting enhanced neural synchrony when practicing FA together.Matiz et al. (2021)conducted a study with randomly assigned dyads of novice meditators who performed FA and instructed mind wandering while either in the same room or different rooms. The results revealed a task effect, where FA was associated with increased gamma activity in the right fronto-limbic region and decreased theta power in the right fronto-parietal junction compared with mind wandering. Furthermore, a task-by-environment interaction was observed, with increased gamma activity in limbic brain areas only when dyads performed FA together in the same room, indicating that the physical presence of a partner during FA enhances inter-subject neural synchrony in limbic brain regions.

## 4 Discussion

Despite considerable research on the neurophysiological mechanisms of FA, clarity regarding the patterns in methodological approaches and key findings within this literature remains limited. This scoping systematic review sought to comprehensively organize and interpret studies using EEG and MEG to investigate FA. Specifically, we compiled information on study population composition, experimental design, and neurophysiological outcomes. Our findings revealed substantial heterogeneity in participant characteristics, which may contribute to the variability observed in neurophysiological outcomes. In contrast, the choice of FA tasks and control conditions was more consistent, although exploring alternative study designs could provide deeper insights into the neurophysiological mechanisms of FA and enhance research rigor. Regarding neurophysiological outcomes, most studies focused on spectral analyses, revealing trends of increased power in the alpha, beta, and gamma bands. Additionally, FA was consistently associated with heightened complexity and reduced criticality measures. Based on our findings, we propose several methodological recommendations to enhance the quality of future research. Notably, we identified significant gaps in the literature, including the limited use of MEG, and the lack of longitudinal studies—particularly during “high-dose” meditation retreats—highlighting important areas for future investigation. This review thus establishes a foundation for the study of the neurophysiology of FA.

### 4.1 Study population

Studies exhibited considerable heterogeneity in their population composition, including variations in sample size, age, and sex, although they were largely consistent in including only healthy participants. Individuals with neuropsychiatric conditions (e.g., depression, anxiety, post-traumatic stress disorder) were noticeably absent from the literature. Further, there was an imbalance regarding sex across studies, with fewer female than male participants, a discrepancy especially pronounced among both novice and long-term meditators. This issue is important to address in future research, given the established associations between sex and electrophysiological brain dynamics (Hernandez et al., 2024;Miraglia et al., 2015). Additionally, most studies failed to report the ethnicities of participants, highlighting a significant gap in demographic reporting. A lack of ethnic diversity is a well-known problem in psychological and neuroscientific research, including meditation studies (DeLuca et al., 2018), making it unclear how brain function during FA might vary across different populations. Future studies should prioritize the inclusion of female participants and those from diverse ethnic backgrounds to directly assess whether these demographic factors influence neurophysiological outcomes during FA, thereby improving the generalizability of findings.

Another potential source of variability among meditators is a history of psychological trauma. Emerging research suggests that individuals with trauma histories may experience unique challenges during meditation (e.g.,Lindahl et al., 2017;Treleaven, 2018), which could be reflected in neurophysiological outcomes. This may be particularly relevant for meditation-naïve and novice practitioners, as early meditation experiences could differ substantially depending on trauma history. However, none of the included studies reported participant trauma history, precluding our ability to assess its impact in this review. Future studies should systematically report trauma history to better understand its role in shaping meditation-related neurophysiological outcomes and influencing the trajectory of meditation proficiency. Additionally, whether long-term meditation practice mitigates the effects of trauma remains an open empirical question that warrants further investigation.

There was also substantial variability in both the amount and type of meditation training among study participants. However, collating the literature on this aspect was challenging due to inconsistent and often incomplete reporting methods. Specifically, studies differed in how they reported participants’ amount of meditation training, using varying metrics, such as total hours versus years of practice, or employing different statistical measures, such as averages versus ranges, to summarize group-level data. In many cases, studies reported minimal or no information regarding the amount of meditation training of participants. Furthermore, the absence of widely accepted criteria resulted in the use of distinct labels and classifications for meditators based on their amount of training or proficiency levels, leading to high variability across studies. Hence, in this review, we classified participants as meditation-naïve, novice meditators, or long-term meditators, based on a combination of reported meditation experience, study author descriptions, participant labels, and recruitment sources. While many studies used the term “expert” to describe participants, we opted for “long-term” as a more accurate reflection of training duration in the absence of direct measures of meditation proficiency. Additionally, there were notable differences in how studies reported the type of meditation training of participants, with some detailing tradition or religious lineage (e.g., Theravada Buddhism) and others identifying particular meditation techniques (e.g., Anapanasati). It is also important to note that most meditation practitioners engage in multiple techniques rather than exclusively practicing FA, raising questions about how varied meditation training influences neurophysiological mechanisms during specific practices. Furthermore, an important but often overlooked factor in meditation research is inter-individual variability in meditation learning. Just as learning curves vary across other cognitive and motor processes, individuals likely differ in how quickly they develop stable attentional control and enter meditative states, with these differences expected to manifest at the neurophysiological level. Neural biomarkers of FA states and reliable bio-behavioural benchmarks of FA skill levels are critical to advancing the field of contemplative neuroscience.

As the field of meditation research continues to grow, developing a standardized approach that outlines best practices for the experimental design and reporting of meditation studies would be beneficial, similar to those established in fields such as neurofeedback (Ros et al., 2020). In particular, future studies should assess and report participants’ meditation training history in a more fine-grained manner to comprehensively capture both the amount and type of training. This would enable investigations into the dose–response effects of meditation training and facilitate examination of how different meditation techniques interact within an individual’s training background to shape neurophysiological outcomes. Additionally, future research should explore inter-individual differences in meditative development using individualized neurophysiological metrics to better characterize the range of responses during FA. Longitudinal studies tracking changes in both behavioural and neural measures could help determine whether certain baseline characteristics predict meditation learning trajectories, ultimately informing more tailored meditation approaches.

We also propose that future studies should emphasize the phenomenological activities of meditation (Sparby & Sacchet, 2022). This approach promises to meaningfully capture key similarities and differences between various practices and avoid confusion generated by the inconsistent and variable use of meditation-related terminology. Adopting a phenomenological approach would also provide a powerful foundation for the study of advanced meditation states and stages that unfold with time and mastery, moving beyond classification based solely on cumulative training duration (Sparby & Sacchet, 2022). Relatedly, neurophysiological metrics may offer an empirical approach to assessing meditation proficiency and tracking meditative progression in a way that reflects the skill-based nature of meditation. Longitudinal studies, particularly those involving intensive retreats, could be especially valuable for identifying neural signatures of meditative proficiency (seesection 4.4*Additional evidence gaps*). Moreover, recent efforts have leveraged a deep understanding of the core theories of contemplative traditions to inform scientific inquiry by generating empirically testable psychological and neural models (Wright et al., 2023), suggesting that a similar approach would be beneficial in the study of FA. Ultimately, such novel approaches will not only enhance our understanding of FA but also provide deeper insights into meditative development and endpoints as well (Galante et al., 2023;Chowdhury et al., 2023;van Lutterveld et al., 2024;Sparby & Sacchet, 2024).

### 4.2 Experimental design: FA tasks and control conditions

In contrast to the variability observed in participant characteristics, the studies showed greater similarity in their choice of FA tasks and control conditions. The majority of studies used breath sensations as the object of meditation (the attentional “anchor”), with some incorporating breath counting, while several studies used mantras, and the remainder used various other unique anchors. While the consistent use of breath as a meditation anchor facilitates cross-study comparisons, the range of potential anchors extends far beyond a focus on the breath. For instance, the classical Buddhist system teaches 40 different meditation anchors, many involving the elements as “kasinas” (a candle flame, a bowl of water, a clay disk, etc.) (*Kammaṭṭhāna*) (Kim, 2019). Meditation anchors that are less studied may hold particular significance for advanced concentrative absorption meditation practices, such as Buddhist Jhana meditation. In Jhana meditation, practitioners aim to achieve deep absorption with the meditation object, leading to states of heightened attention, clarity, high energy, effortlessness, and bliss (Sparby, 2019;Sparby & Sacchet, 2024;Yang, Sparby, et al., 2024). Therefore, future studies should investigate a wider range of meditation anchors, as this could provide valuable insights into advanced meditation practices and uncover distinct neurophysiological patterns associated with each.

The choice of control conditions is critical in meditation research. As reported in this review, most studies used a resting state, followed by various externally guided perceptual tasks, instructed mind wandering, paced breathing, and internally directed cognitive tasks. However, the use of a resting state as a control condition can be problematic, as long-term and even novice meditators may naturally enter meditative states during rest periods, even when not explicitly attempting to meditate (Tang et al., 2012). This can complicate the interpretation of results, as the so-called rest condition may not serve as a true baseline but instead involve some degree of meditative activity. Additionally, different control tasks engage distinct neural systems, which may influence neurophysiological outcomes and their interpretations. For instance, comparisons between meditation and a cognitively demanding task may emphasize functional differences in executive networks, whereas comparisons with passive activities, such as watching a movie, may primarily highlight differences in the default mode network. Future studies should carefully consider control task selection based on the specific research question and participant characteristics to ensure meaningful and interpretable condition comparisons.

While most studies implemented a standard experimental design comparing separate FA task and control condition blocks, several studies used an experience sampling paradigm during FA practice to compare periods of focused attention with naturally occurring mind wandering (e.g.,Brandmeyer & Delorme, 2018;van Son et al., 2019). This approach may be particularly beneficial for studying less experienced meditators, as they may struggle to maintain a consistent and sustained state of focused attention throughout an FA task block. Additionally, performance-based measures of focused attention during meditation, such as the Meditation Breath Attention Score (Frewen & Bao, 2022), have been developed and can be used to correlate neurophysiological changes with the level of focused attention achieved during an FA task. Future studies should consider incorporating experience sampling paradigms and performance-based measures of focused attention to more accurately capture the neurophysiological changes associated with FA, particularly among inexperienced meditators. However, a potential limitation of experience sampling is that probing participants during meditation practice may itself disrupt meditative states—an effect that may disproportionally impact novice meditators. To our knowledge, no direct evidence exists on the extent to which experience sampling negatively impacts meditative states, and future research should investigate this potential limitation.

### 4.3 Neurophysiological outcomes

#### 4.3.1 Spectral analyses

In this review, most studies conducted spectral analyses, focusing primarily on spectral power and functional connectivity across delta, theta, alpha, beta, and gamma frequency bands. Collectively, there were far more within-group comparisons than between-group comparisons. The investigation of each frequency band was unequal, with the most comparisons focusing on alpha, followed by theta, beta, gamma, and lastly, delta. Notably, within each comparison type—within-group, between-group, and functional connectivity—a large percentage of findings were non-significant. Several factors may contribute to this, including the use of small sample sizes, which reduces the statistical power to detect significant effects (Button et al., 2013), and the exploratory nature of this novel research area, which lends itself to less targeted analytical approaches. This highlights the critical importance of full, rigorous, and transparent reporting of all relevant statistics and methodologies that will support across-studies quantitative meta-analyses that can compensate for deficient statistical power endemic in single studies. Nevertheless, for the alpha, beta, and gamma bandwidths, the number of comparisons yielding increased power was approximately double that of those yielding decreased power, across both within- and between-group comparisons. Conversely, for the delta bandwidth, nearly all significant comparisons indicated decreased power. For the theta bandwidth, there was a generally balanced proportion of significant increases and decreases in power.

Increased alpha power is the most widely reported neurophysiological change associated with meditation, consistently observed across various proficiency levels and meditation techniques, including FA (for review,Cahn & Polich, 2006;Chiesa & Serretti, 2010;Fell et al., 2010;Lee et al., 2018;Lomas et al., 2015). Alpha oscillations are known to support attentional processes, such as internalized attention and top-down attentional control (Benedek et al., 2011;Cooper et al., 2003;Klimesch et al., 2007), which may explain the observed increase in alpha power during FA. Increased theta power has also been reported as a significant neurophysiological marker of meditation across proficiency levels and techniques (Cahn & Polich, 2006;Chiesa & Serretti, 2010;Fell et al., 2010;D. J. Lee et al., 2018;Lomas et al., 2015), despite mixed observations of this finding in relation to FA in the current review. Notably, some evidence suggests that increased theta power may be more pronounced among experienced practitioners (Lomas et al., 2015). Theta oscillations, especially in the frontal midline, are associated with cognitive functions such as cognitive control and working memory (Cavanagh & Frank, 2014;Klimesch, 1999), highlighting their potential relevance in FA. Moreover, the simultaneous presence of theta and alpha oscillations during meditation practice is thought to reflect a state of relaxed alertness, which is a hallmark of effective meditation (Britton et al., 2014).

Conversely, gamma, beta, and delta bandwidths have been less studied in the meditation literature, and previous reviews have not revealed consistent patterns (Cahn & Polich, 2006;Chiesa & Serretti, 2010;Fell et al., 2010;D. J. Lee et al., 2018;Lomas et al., 2015). Gamma and beta oscillations, which are high-frequency bandwidths, are linked to sensory and information processing that characterize waking states (Başar-Eroglu et al., 1996;Bosman et al., 2014;Bouyer et al., 1981). However, interpreting the significance of increased power in the beta and gamma bandwidths observed in the current review is challenging. Beta oscillations are associated with a wide range of functions, including sensorimotor processing, attention, emotion, and cognitive control (Engel & Fries, 2010;Güntekin et al., 2013;Symons et al., 2016), making their potential role in FA unclear. Additionally, it is increasingly recognized that high-frequency gamma oscillations overlap with the spectral bandwidth of muscle activity, potentially contaminating gamma results in EEG studies that do not adequately eliminate muscle artefacts (Hipp & Siegel, 2013;Muthukumaraswamy, 2013). Finally, low-frequency delta oscillations are commonly associated with deep non-REM sleep (Hofle et al., 1997). Previous studies utilizing non-FA techniques that similarly observed decreased delta power during meditation compared with rest suggested that this may indicate a more wakeful and aroused state (Cahn et al., 2010,^2013^;Wang et al., 2019). However, given the large percentage of non-significant findings, and the fact that a quantitative synthesis of neurophysiological outcomes across studies was beyond the scope of this review, we refrain from drawing firm conclusions about spectral power changes associated with FA. Moreover, several methodological and conceptual factors further complicate the interpretation of power differences across studies, underscoring the need for careful consideration of these limitations.

#### 4.3.2 Methodological and conceptual considerations in spectral analyses

One key consideration in interpreting findings from spectral analyses is that oscillatory activity arises from multiple neural generators, making neural source inference based on direct comparisons between studies challenging. An increase in power within a given frequency band may not necessarily reflect the same underlying neurophysiological process across studies, as different combinations of cortical and subcortical sources may be driving the observed activity. Although many studies in this review analyzed topographic scalp distributions of spectral power changes to distinguish between neural generators, volume conduction effects can obscure these distinctions (Nunez & Srinivasan, 2006). Consequently, conclusions drawn solely based on comparisons of electrode-level power changes may be misleading. Future research may benefit from more widespread adoption of techniques such as current source density mapping, source localization methods, and microstate analysis (e.g.,Matiz et al., 2021;Thomas et al., 2014), to better resolve the neural origins of spectral power changes and improve the interpretability of findings in FA research (for review,Michel & Brunet, 2019;Michel & He, 2019;Michel & Koenig, 2018;Tenke & Kayser, 2012). For instance, past studies using source localization in meditation research have shown that increased alpha power during FA may originate from somatosensory cortices, rather than frontal executive regions (e.g.,Kerr et al., 2011), while others have linked certain meditative styles (i.e., zazen) to distributed default-mode network regions via low-resolution brain electromagnetic tomography (LORETA) (Faber et al., 2015). While these methods have provided insights into neural sources of oscillatory activity in meditation, future studies should aim to integrate multiple source localization approaches, such as combining LORETA with EEG microstate analysis, to elucidate the neural origins of spectral power changes in FA.

Another important consideration involves the standard approaches used to analyze EEG/MEG power spectra, including those employed by most studies in this review. Traditional methods rely on canonical frequency bands that presume that spectral power changes necessarily reflect shifting oscillatory activity, yet this overlooks other potential contributing factors. For instance, while most studies in this review used fixed frequency ranges (e.g., 8–12 Hz for alpha), individuals naturally vary in their peak oscillatory frequencies. Without accounting for these variations, an observed increase or decrease in power within a given frequency band may instead reflect a shift in peak frequency rather than a true change in power (Donoghue et al., 2020). Future studies should consider applying methods such as empirical mode decomposition (EMD) (Huang et al., 1998) or spectral parameterization models such as fitting oscillations and one-over-f (FOOF) noise modeling (Donoghue et al., 2020) to better isolate true oscillatory changes from individualized peak frequency shifts.

Another factor that is often overlooked in equating spectral power changes with oscillatory activity is the contribution of aperiodic activity (1/*f*)—a dynamic signal component with notable demographic, cognitive, and clinical correlates, as well as physiological relevance (Donoghue et al., 2020). Given its potential influence on spectral features, aperiodic activity should be explicitly parameterized and analyzed (Donoghue et al., 2020). Explicitly modeling the aperiodic component can help distinguish whether increased power within a specific frequency band (e.g., alpha) during FA reflects enhanced neural synchrony or rather a broadband shift due to underlying cognitive state changes. To improve accuracy in spectral analyses—and avoid conflating broadband shifts with true oscillatory activity—future FA research should explicitly model the aperiodic component using spectral decomposition techniques such as FOOOF or IRASA (Irregular Resampling Auto-Spectral Analysis) (Gerster et al., 2022). Lastly, variability in frequency band definitions across studies may contribute to inconsistencies in reported findings (seeSection 4.5*Limitations of the scoping review*).

Furthermore, as previously discussed, there is considerable methodological heterogeneity across studies, complicating the feasibility of conducting a quantitative meta-analysis of spectral power in relation to FA. However, given the large number of studies investigating spectral power during FA, a meta-analysis could be highly beneficial in elucidating these associations more conclusively. To address this heterogeneity, a future meta-analysis could group findings from similar comparison types—for example, based on participants’ level of meditation proficiency, the type of FA anchor, and/or the type of control condition—to examine spectral changes associated with FA more precisely. Notably, our approach of tallying statistically significant spectral power changes across studies has inherent limitations, as it does not account for effect sizes or methodological variations. Critically, most studies included in this review lacked complete spectral power data, often reporting only statistical test results without providing group means, standard deviations, or electrode-level values. This reporting gap precludes the possibility of a meta-analysis based on published data alone and underscores the need for future studies to adopt more comprehensive and standardized reporting practices. Specifically, we recommend that future studies should include electrode-by-electrode means and standard deviations across frequency bands in supplementary materials to facilitate quantitative synthesis. While improving reporting standards is essential for future meta-analyses, inconsistencies in EEG preprocessing pipelines and spectral estimation methods also present a significant challenge for cross-study comparability. Establishing community-wide guidelines for spectral data sharing, similar to BIDS-EEG (Pernet et al., 2019), would help mitigate these discrepancies. Indeed, making raw data and preprocessing pipelines publicly available will help to enhance the reproducibility and comparability of spectral power findings in FA research.

#### 4.3.3 Other neurophysiological outcomes

In this review, a minority of studies investigated non-linear measures, ERPs, and machine-learning classification methods, with highly varied specific outcome measures among each. Given that the brain operates as a complex and chaotic system (Breakspear, 2017;Chialvo, 2010;McKenna et al., 1994;Sporns, 2022), non-linear dynamical systems approaches—including measures of complexity and criticality—can provide complementary insights into neural dynamics that extend beyond conventional spectral analysis (Lau et al., 2022;Pereda et al., 2005;Pritchard & Duke, 1992). Measures of complexity indicate the diversity and integration of multi-scale neural interactions, reflecting the brain’s ability to process information efficiently and adaptively (Bassett & Gazzaniga, 2011;Sporns, 2022). Criticality refers to a neural system operating at the edge between randomness and orderliness (Cocchi et al., 2017;Heiney et al., 2021), the point at which it is most capable of rapid and flexible reorganization (Deco et al., 2013;O’Byrne & Jerbi, 2022;Shew & Plenz, 2013;Singer, 2013;Tognoli & Kelso, 2014). With the exception of one between-group comparison (Kakumanu et al., 2018), this review observed a consistent trend of increased complexity and decreased criticality measures associated with FA versus control conditions and other meditation techniques, or as a function of the amount of meditation training of participants.

Complexity and criticality are often treated as equivalent concepts in the literature. However, in addition to observations of their divergence across FA studies included in this review,Walter & Hinterberger (2022)found strong negative correlations between complexity and criticality measures within a single study, which together support the notion that these measures may represent distinct aspects of brain dynamics. Notably, a previous systematic review observed a similar trend of increased complexity during meditation of all types, where the effect was most prominent among long-term meditators (Atad et al., 2023). Increased complexity has also been observed during other altered states of consciousness, such as the psychedelic state (for review,Girn et al., 2023;McCulloch et al., 2022), and has been interpreted, under predictive processing principles, as a shift towards increased “bottom-up” information flow in conjunction with a relaxation of high-level priors (Laukkonen et al., 2023;Laukkonen & Slagter, 2021;Atad et al., 2023). This aligns with the aim of FA to shift attentional focus away from thoughts, abstractions, and concepts, and instead focus on present moment sensory experiences. Decreased criticality has been interpreted in the presently reviewed literature as a shift towards a subcritical regime, characterized by stabler and less responsive brain activity (Irrmischer et al., 2018;Walter & Hinterberger, 2022). In the context of FA, this may correspond to stabilized attention and reduced distractions, explaining why decreased criticality was particularly evident among long-term meditators.

Several studies investigated auditory and visual attention during FA by examining a range of ERP components—P1, P2, P3, N1, N2, MMN, LFN, ERN, Pe, and LPP—which correspond to specific neural responses to stimuli during the FA task. ERPs are average electrical potentials generated by groups of neurons in response to a stimulus, such as an auditory tone or visual cue, represented by waveforms that comprise a series of positive and negative peaks named according to their position in the series (e.g., P1 is the first positive peak, N1 is the first negative peak) (Luck et al., 2000). Other ERPs that were frequently examined in this literature include the LFN, a slower latency negative response that is thought to reflect attentional monitoring of the sensory environment (Fucci et al., 2018), and the MMN, a well-characterized component elicited by a sudden change in repeating auditory stimuli that is thought to underlie an implicit perceptual learning process (Garrido et al., 2009;Näätänen et al., 2007). This review found no consistent pattern in the reported changes in ERP component amplitudes during FA. However, given the limited number of studies and considerable variability in study tasks and examined ERP components, further research is necessary to achieve more definitive insights into auditory-evoked and visually evoked neural responses during FA. Additionally, as with experience sampling, a potential limitation of ERP studies is that stimulus events could interfere with meditative states. However, to our knowledge, no direct evidence has assessed the extent of this disruption, and future research should investigate this potential limitation. Other studies used various machine-learning classification methods, such as multivariate random forest classifiers, support vector machines, and linear discriminant analysis, to distinguish neurophysiological patterns based on participants’ amount of meditation training, differentiate between FA and OM techniques, and detect episodes of mind wandering during FA. Despite variability in the classification methods and outcomes, all studies included in this review achieved high classification accuracies, supporting the idea that FA may be associated with consistent and specific neurophysiological patterns.

Finally, several studies reported outcomes that could not be grouped with those previously mentioned due to methodological heterogeneity. These include studies using consumer-grade EEG devices, which often reported idiosyncratic metrics such as a “meditation score” or the percentage of time spent in a “calm state.” These devices typically have low-density sensor arrays (1–16 channels), limiting spatial resolution and localization accuracy, and require further research validation (Sabio et al., 2024). Despite these limitations, consumer-grade EEG devices offer the significant advantage of enabling a greater scale of neurophysiological data collection in naturalistic settings. Future studies should aim to conduct standard spectral power analyses with these devices instead of relying on opaque metrics that are unique to each consumer-grade EEG device. This approach could extend meditation research beyond controlled laboratory environments, providing valuable insights into real-world practices.

Additionally, two studies utilized hyperscanning designs among dyads to compare joint FA practice with individual practice. Due to the unique nature of this experimental design, we grouped findings from these studies separately. Notably, both hyperscanning studies observed enhanced neural synchrony during joint FA practice compared with individual practice. Despite few studies in this review pursuing this approach, hyperscanning has shown to be a reliable and highly promising technique that has illuminated the inter-brain neural underpinnings of social interaction across myriad domains (Czeszumski et al., 2020), and thus may also be useful in the context of meditation. Specifically, hyperscanning may help demonstrate the neurophysiological patterns uniquely associated with joint meditation practice, or the “sangha effect,” which is widely recognized as a critical component of the meditative path (e.g.,Hanh, 2010). Future research should further leverage this innovative technique to examine how joint meditation influences inter-brain dynamics, as few studies to date have investigated this phenomenon. Additionally, exploration of the potential neural mechanisms underlying increased inter-individual synchrony during joint meditation is warranted.

In summary, this scoping review has provided a comprehensive overview of the existing literature, specifically focusing on study population, experimental design, and neurophysiological outcomes. Across each of these domains, we have identified several evidence gaps and made recommendations for future research. However, additional evidence gaps remain when considering the whole body of existing studies, which warrant further discussion.

### 4.4 Additional evidence gaps

Although this review was inclusive of both EEG and MEG studies, it is notable that only four studies, based on two independent datasets, used MEG during FA (Calvetti et al., 2021;D’Andrea et al., 2024;Heinilä et al., 2024;Marzetti et al., 2014). While both EEG and MEG provide non-invasive measurements of neuronal population excitability with high temporal resolution, MEG offers specific strengths that could further advance the field. Notably, MEG is known to provide superior spatial resolution compared with EEG, allowing for improved localization of the generators of neural activity (Hedrich et al., 2017;Vorwerk et al., 2014), potentially achieving up to millimeter-scale accuracy (Troebinger et al., 2014). Additionally, MEG produces a higher signal-to-noise ratio than EEG, particularly for gamma band activity (Lopes da Silva, 2013). This advantage is particularly helpful considering the increasingly well-recognized overlap between the spectral bandwidth of gamma oscillations and muscle artefacts, as previously discussed. Moreover, advanced MEG applications allow for the mapping of neurophysiological patterns to large-scale brain networks, closely resembling those delineated using fMRI, such as the default mode network (Brookes et al., 2011). Such applications have provided novel insights into the context of psychiatric illness (Alamian et al., 2017;Sunaga et al., 2020) and could be similarly applied to the study of FA.

With only a few exceptions (Duda et al., 2023;Irrmischer et al., 2018;Jo et al., 2019;Rodriguez-Larios et al., 2024;Saggar et al., 2012;Steinhubl et al., 2015), the existing literature predominantly relied on cross-sectional comparisons, which makes it difficult to draw conclusions about the causal role of meditative practice on reported neurophysiological outcomes. Therefore, the use of longitudinal study design is necessary to rule out factors unrelated to meditation training, such as variability in attentional effort and motivation (Jensen et al., 2012). Also, longitudinal studies can provide valuable insights into how these neurophysiological changes develop over the course of meditation training, potentially identifying critical periods and thresholds of practice that are most influential in encouraging meditative development and achieving endpoints (Chowdhury et al., 2023;Galante et al., 2023;van Lutterveld et al., 2024). Studies involving intensive meditation retreats may be particularly fruitful in this regard, as they can track changes over defined, concentrated periods of practice (King et al., 2019). Such designs would not only help establish causal relationships but also shed light on neurophysiological dynamics related to advanced meditative states that are typically only accessible from long-term, intensive, and directed FA meditation practice (Sparby, 2019;Sparby & Sacchet, 2024;Yang, Sparby, et al., 2024).

Additionally, none of the included studies evaluated the effects of expectations on neurophysiological outcomes, and the potential role of expectancy effects remains largely unexplored in neuroimaging meditation research more broadly. However, some studies have examined expectancy effects on meditative engagement, cognitive task performance, and clinical outcomes, with mixed findings (Ghanbari Noshari et al., 2023;Haddad et al., 2020;Hicks et al., 2016;Prätzlich et al., 2016). Given the potential influence of expectations on meditative experience and associated neurophysiological outcomes, future neuroimaging studies on meditation should account for expectancy effects to better isolate the specific contributions of meditation practice itself. Relatedly, an additional source of variability in meditation research is the socio-cultural framing of different meditation techniques. Each tradition presents its practices within distinct conceptual and philosophical frameworks, which may influence practitioner expectations, the engagement of specific mental processes, and, ultimately, neurophysiological outcomes. Taken together, future research that addresses these critical evidence gaps may help to provide a deeper understanding of the neurophysiological mechanisms associated with FA.

### 4.5 Limitations of the scoping review

While this scoping review comprehensively collates the existing literature on the neurophysiology of FA, we note several limitations. Given the variability in how studies reported meditation experience and the absence of standardized guidelines, our classification of meditators was necessarily subjective, relying on reported meditation experience, study author descriptions, participant labels, and recruitment sources. Another limitation is the substantial variability in the definitions of frequency bands across the included studies. Although most studies used frequency band definitions that aligned with commonly employed ranges, there was often variability in the high and low cutoffs of the frequency band ranges. Additionally, in some cases, the traditional frequency bands were further divided into sub-bands (e.g., low alpha, high alpha). To collate spectral analyses findings, we categorized results based on the definitions used by each study and combined results across sub-bands where applicable. This approach may have oversimplified the data and obscured specific patterns within frequency sub-bands. Another notable limitation is the extensive variability in data acquisition and processing methods among the included studies. Although we extracted data related to the characteristics of EEG/MEG devices, sampling rates, filtering techniques, and data exclusion methods, this information was so variable that it was impossible to collate it effectively. This methodological heterogeneity may affect the consistency and comparability of observed neurophysiological outcomes. Additionally, this review may be influenced by a positive results bias, as it included only published studies, which may yield an overrepresentation of positive or significant findings. Relatedly, the possibility that included studies disproportionately reported comparisons yielding significant as opposed to null findings must be considered.

## 5 Conclusion

This scoping systematic review offers a comprehensive overview of the current literature investigating the neurophysiological mechanisms of FA using EEG and MEG. We collated key study characteristics, including population composition, experimental design, and neurophysiological outcomes. Our findings revealed significant heterogeneity in participant characteristics, which may contribute to variability in the reported neurophysiological outcomes. In contrast, the choice of FA tasks and control conditions was largely consistent. Based on these findings, we proposed several methodological recommendations for future research: increasing the inclusion of female participants, developing a standardized approach that outlines best practices in experimental design, and reporting of meditation studies, employing phenomenologically based classifications of meditation techniques, exploring a broader range of meditation anchors, and ensuring the careful selection of appropriate control conditions, among several others. In terms of neurophysiological outcomes, most studies focused on spectral analyses, revealing trends of increased power in the alpha, beta, and gamma bands. However, a future meta-analysis—enabled by more comprehensive reporting of group-level data—is necessary to further clarify these associations and provide more definitive insights. Other outcomes, such as non-linear analyses, ERPs, and machine-learning classification, were examined in only a minority of studies. Nevertheless, a consistent trend of increased complexity and decreased criticality measures associated with FA was observed. Furthermore, the limited use of MEG and the lack of longitudinal designs, particularly those involving intensive meditation retreats, comprise significant evidence gaps in the literature and thus represent important opportunities for future research. In conclusion, this review provides a strong foundation for the study of FA’s neurophysiology, as well as the study of advanced meditation (Sacchet et al., 2024;Wright et al., 2023) and neuroscience-informed meditative development (Abellaneda-Pérez et al., 2024).

## Data and Code Availability

All key data extracted from the included studies are fully presented in this manuscript, either in tables or described within the text.

## Author Contributions

Jonathan M. Lieberman: Conceptualization, Methodology, Investigation, Data Curation, Writing—Original Draft, Writing—Reviewing and Editing, and Visualization. Patrick A. McConnell: Conceptualization, Methodology, Investigation, Writing—Original Draft, Writing—Reviewing and Editing. Mar Estarellas: Investigation, Data Curation, Writing—Reviewing and Editing. Matthew D. Sacchet: Conceptualization, Methodology, Writing—Reviewing and Editing, Supervision.

## Funding

Jonathan M. Lieberman has received funding support from the Canadian Institute of Health Research (CIHR) (Funding Reference Number 187470). Patrick A. McConnell is supported by the National Institute of Drug Abuse (NIDA) (Grant Number T32DA007250). Matthew D. Sacchet and the Meditation Research Program are supported by the National Institute of Mental Health (Project Number R01MH125850), Dimension Giving Fund, Tan Teo Charitable Foundation, and additional individual donors.

## Ethics Statement

This article does not contain original research.

## Declaration of Competing Interest

The authors declare that the research was conducted in the absence of any commercial or financial relationships that could be construed as a potential conflict of interest.
